# Lessons Learned on Obtaining Reliable Dynamic Properties for Ionic Liquids

**DOI:** 10.1002/cphc.202401048

**Published:** 2025-02-18

**Authors:** Tom Frömbgen, Paul Zaby, Vahideh Alizadeh, Juarez L. F. Da Silva, Barbara Kirchner, Tuanan C. Lourenço

**Affiliations:** ^1^ Mulliken Center for Theoretical Chemistry University of Bonn Beringstraße 4 D-53115 Bonn Germany; ^2^ São Carlos Institute of Chemistry University of São Paulo P.O. Box 780 13560-970 São Carlos SP Brazil

**Keywords:** Ionic liquids, Molecular dynamics, Computational chemistry, Ion pairs, Electrochemistry

## Abstract

Ionic liquids are nowadays investigated with respect to their use as electrolytes for high‐performance energy storage materials. In this study, we provide a tutorial on how to calculate dynamic properties such as self‐diffusion coefficients, ionic conductivities, transference numbers, as well as ion pair and ion cage dynamics, that all play a role in judging the applicability of ionic liquids as electrolytes. For the case of the ionic liquid [C2C1Im][NTf2] , we investigate the performance of different force fields. Amongst them are non‐polarizable models employing unity charges, a charge‐scaled version of a non‐polarizable model, a polarizable model and another non‐polarizable model with refined Lennard‐Jones parameters. We also study the influence of the system size on the dynamic properties. While all studied force field models capture qualitatively correct trends, only the polarizable force field and the non‐polarizable force field with refined Lennard‐Jones parameters provide quantitative agreement to reference data, making the latter model very attractive for the reason of lower computational costs.

## Graphical abstract

The performance of different force fields in the calculation of transport properties of ionic liquids is studied at the example of [C_2_C_1_Im][NTf_2_]. Qualitatively, all investigated force field formulations yield accurate results, but to achieve quantitative agreement to reference data, either explicit polarization or a refinement of the classical Lennard‐Jones parameters have to be taken into account.

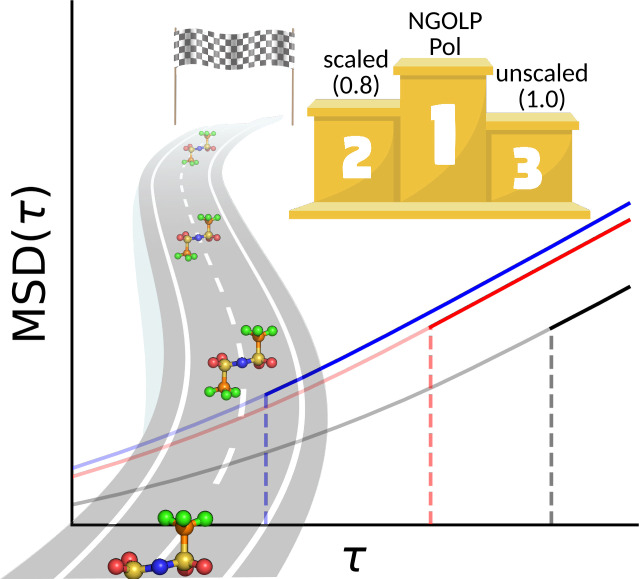

## Introduction

Ionic liquids (ILs) are a versatile class of materials with applications ranging from extraction processes[^1^, ^2^] to electrolytes[^3^, ^4^] and lubricants. This is due to their characteristic physicochemical properties[^5^, ^6^] of ILs, such as low vapor pressure,^[7]^ relatively low toxicity,^[8]^ wide electrochemical window and high thermal stability,[^9^, ^10^] and reasonable ionic conductivity.^[11]^

While in the beginning of the ionic liquids era, static properties have been the focus of scientific investigations first, the study of their dynamic properties followed shortly after and is well established today.[^6^, ^12^, ^13^, ^14^, ^15^, ^16^, ^17^] In this context, molecular dynamics (MD) simulations based on force fields (FFs) have been widely used because of their ability to elucidate the structure and dynamics of the ions from an atomistic point of view.[^18^, ^19^, ^20^] For example, MD simulations give access to several transport properties, such as viscosity, ionic conductivity, and diffusivity. However, the high viscosity and slow dynamics of the ILs pose challenges in the calculations of these properties, sometimes leading to large deviations with respect to the experimental data.

The atomic charges used in nonpolarizable force fields are mostly obtained from *ab initio* calculations for isolated ions, often overestimating the charge magnitude and the strength of electrostatic interactions in MD simulations, and consequently reducing ion dynamics. Moreover, the lack of description of charge transfer and polarizability in non‐polarizable force fields also contributes to the overestimation of viscosity.^[21]^ Some approaches have been developed to overcome these challenges, such as (i) scaling the atomic charges by empirical factors, generally between 0.70 and 0.80,[^22^, ^23^, ^24^, ^25^, ^26^, ^27^, ^28^, ^29^] (ii) atomic charges obtained from bulk phase calculations,[^30^, ^31^] and (iii) polarizable force fields.[^21^, ^32^, ^33^, ^34^, ^35^]

The scaling of atomic charges by empirical factors reduces Coulomb interactions between the ions, and consequently, speeds up the dynamics and improves the correlation between the MD and the experimental data.^[36]^ However, this approach can also lead to a poor description of the system density and may impact the local structure of the ions adversely, especially when mixtures are considered.^[37]^ Applying scaling factors in the range of 0.70 and 0.80 is based on the determination of atomic charges using ionic pairs i. e., a cation and anion together, in gas‐phase calculations using Density Functional Theory (DFT).[^38^, ^39^, ^40^, ^41^] Leontyev and Stuchebrukhov,^[42]^ justify scaling the charges, based on the dielectric constant. For most ILs, the total charge for the ions is found to be in the range of ±0.70 to ±0.80 . At the same time, these scaling factors provide a good balance between improving the transport properties and compromising the density and structure of the system. A more detailed discussion on force fields with scaled charges was recently provided by Jorge.^[43]^

Recently, a new approach has been introduced to obtain the atomic charges for ILs and similar systems.[^44^, ^45^] Therein, the charges are calculated employing a self‐consistent MD/DFT scheme.^[46]^ FF‐based MD simulations are used to provide the equilibrated bulk structure for an IL and subsequently, a periodic DFT calculation is performed on an MD snapshot, during which the atomic charges are determined. From that point on, new MD simulations are carried out and, after equilibration, the bulk phase atomic charges are again determined by means of a DFT calculation. This routine is repeated until the atomic charges converge within a tolerance criterion. As noted, in general, the total charges for ions using this approach are within ± 0.70 to ± 0.80, which impacts the transport properties similarly to the aforementioned charge‐scaling approach. However, since the charges are obtained in the bulk phase, the final (electronic and geometric) structure can provide important insights regarding charge transfer and polarizability effects in the condensed phase.

In the last decade, polarizable force fields have become widely used in MD simulations of IL‐based systems, mainly due to increased computational power and the development of new force field models, such as CL&Pol,[^32^, ^47^] APPLEP,[^48^, ^49^] CHARMM polarizable,^[50]^ a combination of CL&P with the polarizable ion model,[^51^, ^52^] AMOEBA‐IL,[^53^, ^54^] a GAFF‐based model by Wang and Maginn^[35]^ and others. These force fields can properly describe electronic polarization and charge effects using different methodologies, such as fluctuating charges,^[55]^ Drude oscillators,^[56]^ or induced point dipoles.^[57]^ Generally, polarizable force fields yield better correlations between the transport properties obtained from MD simulations and those measured experimentally.^[35]^ Unlike scaling of atomic charges, the addition of explicit polarization in the force fields does not negatively affect system densities or local ion structures.^[37]^ However, this improvement comes with notably increased computational costs, which makes the production of many replicas or long simulations less feasible and thus indirectly leads to reduced statistical sampling.

Beyond the approaches highlighted above, it is also possible to improve the description of the transport properties in classical force fields by adjusting/refining the Lennard‐Jones parameters to match experimental data or *ab initio* calculations.[^27^, ^58^, ^59^, ^60^, ^61^] For example, Köddermann *et al*.^[58]^ empirically adjusted the σ and ϵ parameters of ${\left[{\mathrm{NTf}}_{2}\right]}^{-}$ taken from the CL&P force field^[62]^ to match the experimental data for density, self‐diffusion coefficients, and rotational relaxation times. By doing that, they obtained very good agreement to experimental transport properties without scaling the atomic charges and sacrificing densities and other structural properties. Later, this force field was updated and became the NGOLP force field,^[59]^ which is discussed in more detail below. Recently, Chaumount *et al*.^[27]^ followed a similar approach and adapted the LJ parameters for some deep eutectic solvents. Moreover, Balasubramanian and coworkers combined the refinement of Lennard‐Jones parameters and atomic charges obtained by the self‐consistent MD/DFT scheme for certain ILs.[^60^, ^61^] All the above examples show that the refinement of Lennard‐Jones parameters can also be used to improve the ILs transport properties in MD simulations without changing the description of atomic charges in the system.

We see a demand to systematically assess the impact of the use of different non‐polarizable and polarizable force fields in the calculation of IL transport properties and how to improve their reliability. Therefore, we provide a tutorial on how to improve the reliability of calculating dynamics properties of ILs, and to decrease the user bias in the search for linear regimes of the mean square displacements and in the fitting procedure of nonlinear properties. In this context, we acknowledge the excellent article by Maginn *et al*.^[63]^ that “discusses the best practices that should be followed to ensure that the simulation output is reliable” in view of the calculation of transport properties.

In the following, a comprehensive overview of the methodology employed in this study is provided, including relevant computational details and a tutorial, followed by a presentation and discussion of the results.

## Theoretical Approach and Computational Details

**Figure 1:**
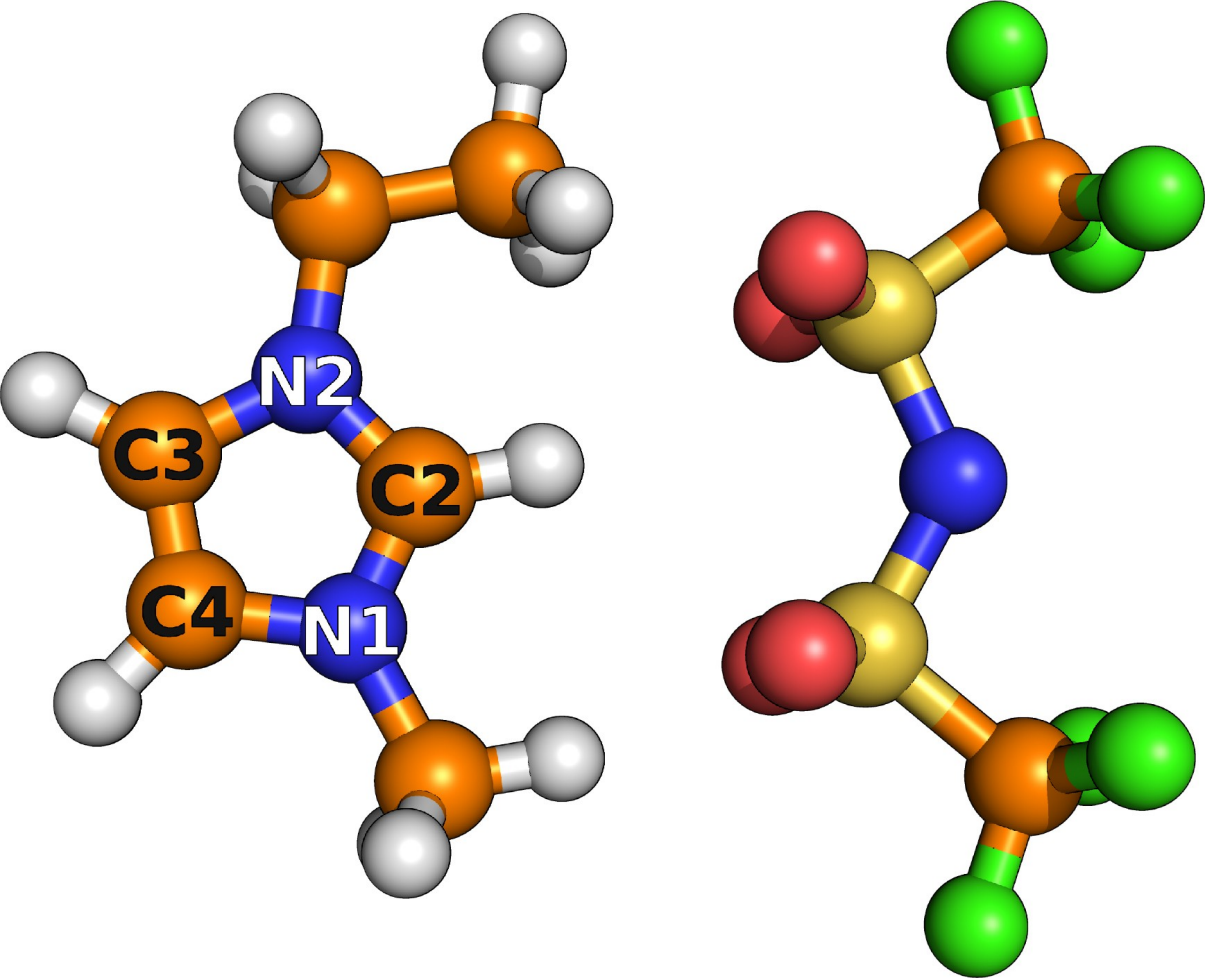
Ball‐and‐stick representation of the 1‐ethyl‐3‐methylimidazolium cation, [C2C1Im]+ , and the bis(trifluoromethylsulfonyl)imide anion, ${[\mathrm{NTf}}_{2}{]}^{-}$ , with labeled atoms that are referenced within this work. The color code is orange: carbon, blue: nitrogen, light gray: hydrogen, red: oxygen, gold: sulfur, green: fluorine.

In order to develop our workflows for calculating the transport properties of ionic liquids, we applied three different charge models to describe the IL 1‐ethyl‐3‐methylimidazolium bis(trifluoromethylsulfonyl)imide, [C2C1Im][NTf2] , shown in Figure 1. In particular, we will explore the following charge models: (i) total integer charges for the ions, i. e., ± 1.0, (ii) total integer charges scaled by a factor of 0.80, and (iii) the addition of explicit polarizabilities using Drude particles. We selected the most common transport properties, namely self‐diffusion coefficients, ionic conductivity, ideal transference numbers, ion‐pair/cage lifetimes and rotational dynamics.

To ensure the robustness of the calculated data, we also propose a refined method to calculate self‐diffusion coefficients and ionic conductivity from Einstein‐based methods by reducing the user bias and other error sources. Simulation box size effects were taken into account in the calculation of dynamic properties. In the following, we provide all technical details regarding the force field models and the computational details for the simulations and strategies used in the transport properties calculations.

### Force Field Models

We used the CL&P force field for some of the classical MD calculations, developed by Pádua and Canongia Lopes,[^62^, ^64^, ^65^, ^66^, ^67^, ^68^] which is based on the OPLS force field,^[69]^ but specifically parameterized for common ionic liquids. Furthermore, we considered the NGOLP force field from Neumann *et al*.^[59]^ in which the original Lennard‐Jones and dihedral parameters of ${[\mathrm{NTf}}_{2}{]}^{-}$ from CL&P were refined to better describe experimental densities, self‐diffusion coefficients, and rotational dynamics.^[59]^

For CL&P we considered three datasets; (i) total integer charges for ions, ± 1.0, obtained directly from the CL&P database,^[70]^ (ii) scaled charges, in which the default force field charges were scaled by 0.80 and (iii) CL&Pol,[^32^, ^47^] which is the polarizable version of the CL&P force field using Drude particles^[56]^ attached to non‐hydrogen atoms. The NGOLP force field was used as its default version, that is, ± 1.0 total integer charges for the ions. A detailed overview of the charges used in the force fields is provided in Section 1 of the Supporting Information, and exemplary input files of the simulations can be found in a data repository.

### Molecular Dynamics Simulation Details

The initial configurations for the MD simulations were created using the molecular structures provided in the CL&P database as well as the fftool^[70]^ and PACKMOL software packages,^[71]^ where the ion pairs were randomly packed in a cubic box following the compositions shown in Table 1. Beyond the effect of the force field parameters, we also investigated size effects regarding the calculations of the transport properties. Therefore, for the integer charge CL&P simulations, three different system sizes were investigated, namely 256, 512 and 1024 ion pairs (labeled MD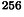 , MD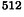 and MD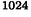 ). For CL&Pol only 256 and 512 ion pairs were used (polMD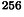 and polMD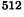 ). For the CL&P force field with charges scaled by 0.8 and the NGOLP force field, we considered only a system size of 512 ion pairs, which were labeled as MD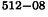 and NGOLP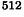 , respectively. An overview of the systems and their corresponding labels can be found in Table 1.

**Table 1:** Overview of the studied systems: system labels, number of ion pairs N , and employed force fields.

Label	N	Ref.
MD 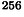	256	CL&P
MD 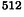	512	CL&P
MD 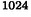	1024	CL&P
MD 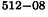	512	CL&P
NGOLP 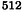	512	NGOLP
polMD 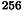	256	CL&Pol
polMD 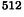	512	CL&Pol

All nonpolarizable simulations were performed using a six‐step approach:

1. Energy minimization using the conjugated‐gradient method to remove energy hot spots.
2. NVE ensemble equilibration for 100 ps.
3. NpT ensemble equilibration at 1.0 atm and 340 K for 5 ns.
4. Compression of the box to the average volume of the last 2 ns of the previous NpT equilibration within 0.5 ns in the NVT ensemble.
5. NVT ensemble equilibration of 2 ns at 340 K.
6. NVT production run of 30 ns with a dumping frequency of 1 ps.

For the polarizable MD simulations, we used a simplified approach for the simulations, which is justified by the higher computational costs of the polarizable force field. Therefore, for all the polarizable MD simulations, we applied the following approach:

1. Energy minimization, as in nonpolarizable simulation.
2. NVT ensemble equilibration for 2 ns at 340 K.
3. NpT ensemble equilibration at 1.0 atm and 340 K for 5 ns.
4. NVT production run of 30 ns with a dumping frequency of 1 ps.

To improve statistical sampling in this study, we performed four additional NVT production runs of 31 ns for all systems using the initial configurations of the first production runs with changed initial velocity seeds, where only the last 30 ns were used for analysis. Therefore, the data presented below are the means of five independent trajectories, as recommended, for example, by Coveney and coworkers^[72]^ or Zuckerman and coworkers^[73]^ (see further discussion in the Tutorial Part). The aforementioned procedure was validated by calculating the resulting bulk phase density after equilibration. These were found to be in excellent agreement with the experimentally derived reference ($|\Delta \rho_{\mathrm{ref}}|<1.5\%$ ), except for the charge‐scaled system MD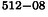 , showing a (negative) deviation of 4.41 %. The latter can be expected as a result of the modification of the force field by scaling the charges and is deemed acceptable. Further details are provided in Section 2 of the Supporting Information.

To integrate the equations of motion, we used the Velocity Verlet algorithm as implemented in LAMMPS version 20Jun2020^[74]^ with a time step of 1 fs. For the Coulombic equations, we used the particle‐particle particle‐mesh solver^[75]^ with an accuracy of e‐5 and a cut‐off of 1.2 nm, while for the short‐range interactions only the cutoff was considered. In nonpolarizable simulations, we controlled temperature and pressure using the Nosé−Hoover methods[^76^, ^77^, ^78^] with coupling times of 0.1 and 1 ps respectively. As stated in the seminal CL&Pol force field work,[^32^, ^47^] to properly handle temperature fluctuations in polarizable simulations, it is necessary to use a dual thermostat, which treats the atoms and Drude particles with two distinct thermostats. Therefore, in the polarizable force field, we applied the Temperature‐Grouped Dual‐Nosé−Hoover method^[79]^ with the same coupling times for the non‐Drude particles and 0.2 ps for the Drude particles, which were maintained at 1 K.

**Figure 2:**
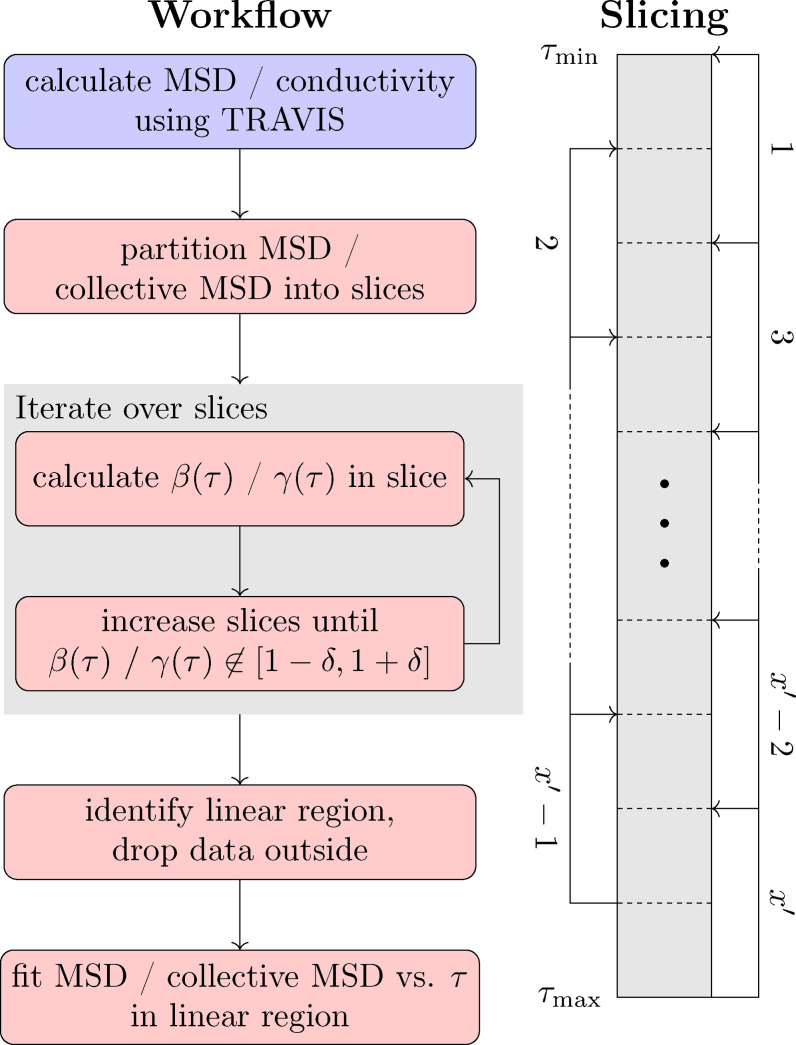
Flowchart representation of the proposed workflow to obtain reliable self‐diffusion and ionic conductivities (left) and illustration of partitioning the MSD / collective MSD data into slices (right). Of the workflow, all steps in steps colored in red are performed within msdiff, our python routine, while the blue step done using TRAVIS (or other software).

**Figure 3:**
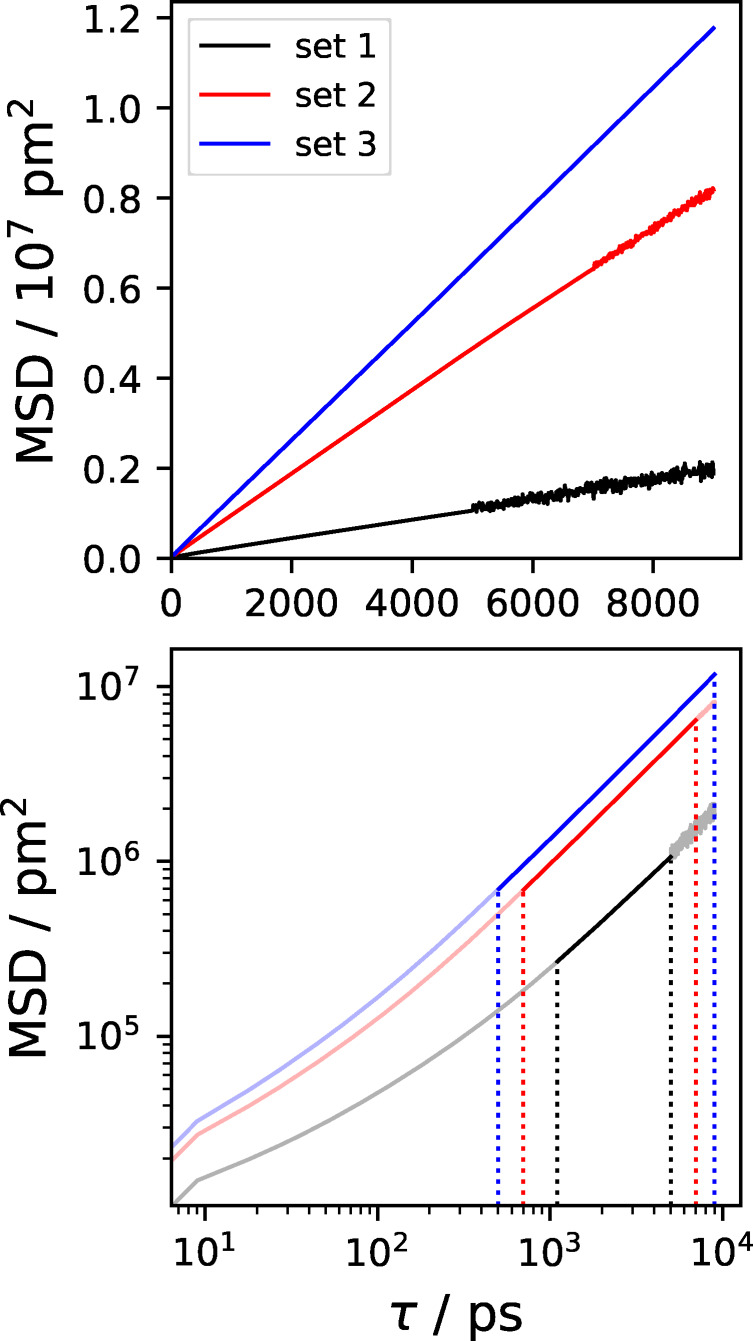
Illustration of the procedure to determine linear regimes in mean squared displacement (MSD) data. Three arbitrary MSD data sets are presented (black, red, and blue lines). Top panel: Linear representation of the MSD as a function of the correlation time τ . Bottom panel: logarithmic representation of MSD (τ ). The identified linear regimes are colored, while the non‐linear regimes are shaded. Vertical dashed lines indicate the start and end of the linear regimes.

**Figure 4:**
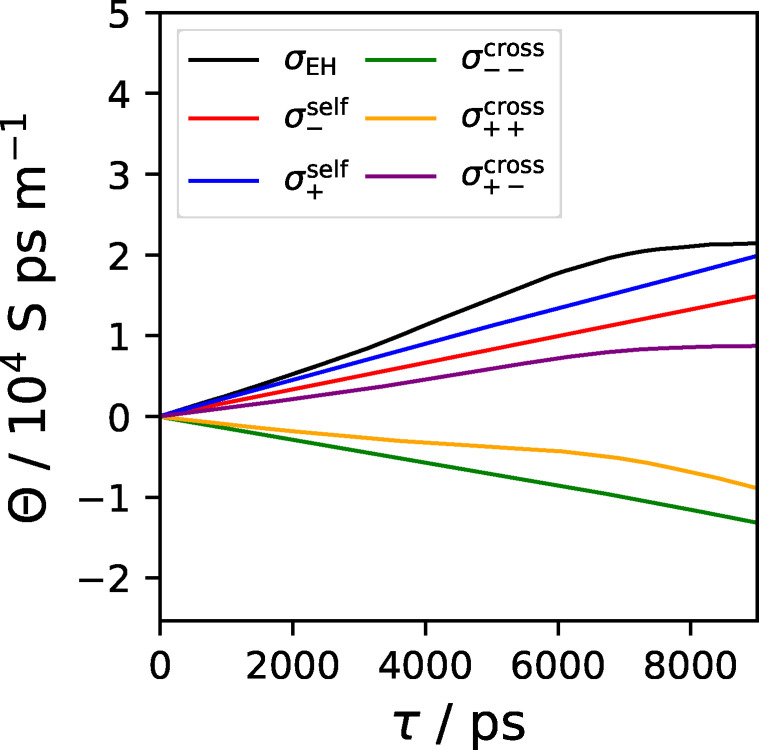
Individual components of the collective MSD (multiplied by the constants shown in Eq. 6) obtained from an arbitrary trajectory. Note that some components, especially the cross‐terms (shown in yellow and violet) are often nonlinear.

**Figure 5:**
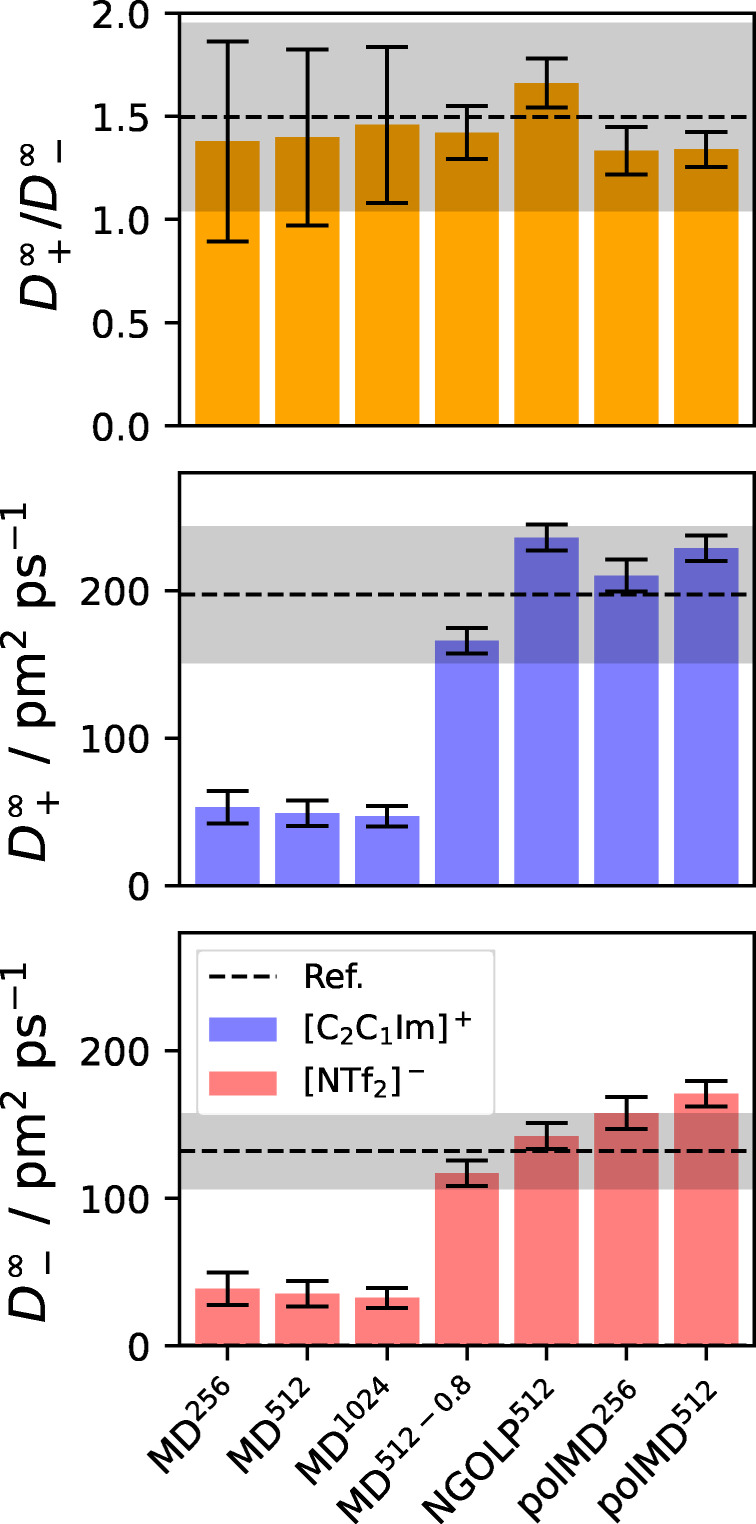
Infinite box size diffusion coefficients from Table 2. Bottom panel: Anion coefficients $D_{-}^{\infty }$ (blue bars). Center panel: Cation coefficients $D_{+}^{\infty }$ (red bars). Top panel: Ratio of cation vs. anion coefficients $D_{+}^{\infty }/D_{-}^{\infty }$ (orange bars). Reference values and their uncertainties, taken from Ref. [106], are shown as black dashed lines and gray shaded horizontal bars, respectively.

**Figure 6:**
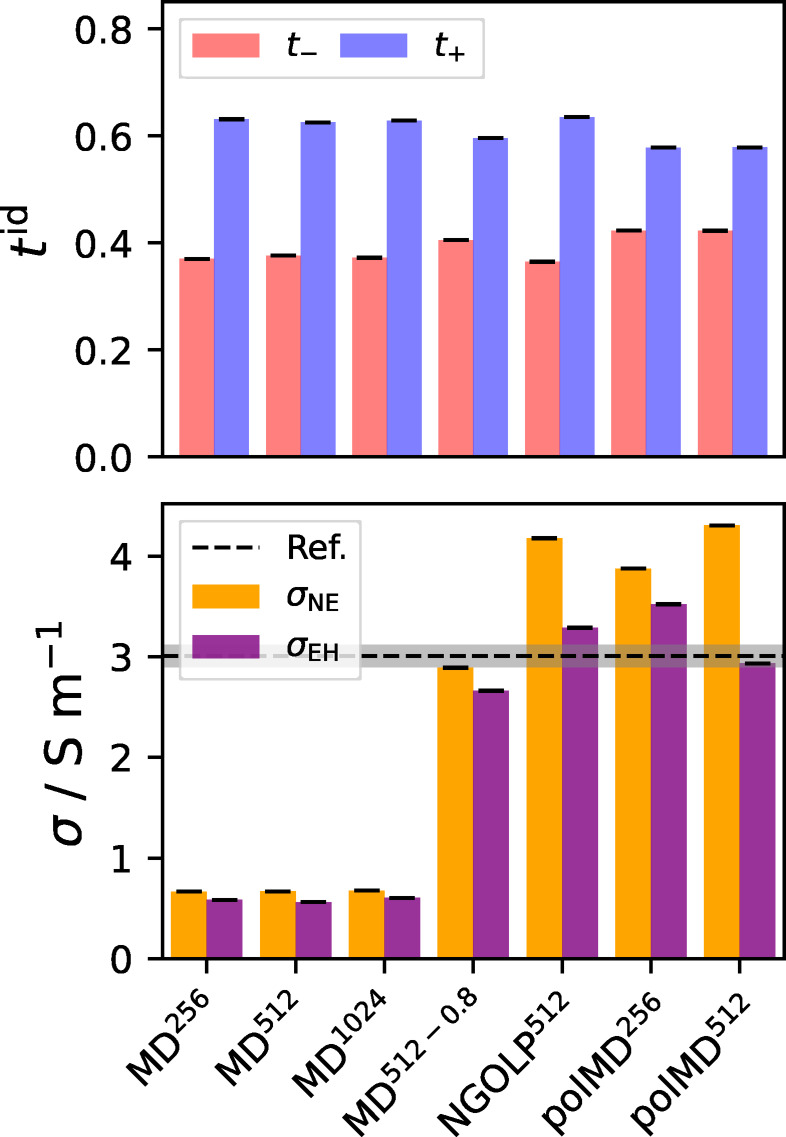
Bottom panel: Nernst−Einstein ionic conductivity $\sigma_{\mathrm{NE}}$ (orange bars) and Einstein−Helfand ionic conductivity $\sigma_{\mathrm{EH}}$ (purple bars), all in $S\;\;m^{-1}$ . Corresponding values are shown in Table 3. The reference value is shown as black dashed line and its uncertainty in gray. Top panel: Ideal transference numbers of cations, $t_{+}$ , (blue bars), and anions, $t_{-}$ (red bars).

All trajectories were analyzed using the TRAVIS package.[^80^, ^81^] However, the routine used to calculate the ionic conductivities by means of the Einstein method (implemented in TRAVIS) will be available to the public in the near future. The Python code msdiff, to calculate self‐diffusion coefficients and conductivities, based on the TRAVIS output, is publicly available on GitHub at https://github.com/kirchners‐manta/msdiff. Lifetimes were calculated by post‐processing outputs from TRAVIS and performing exponential fits using the lmfit library.^[82]^ Furthermore, to ensure the reproducibility of this work, all data and input files relevant to the MD simulations are available on GitHub at https://github.com/kirchners‐manta/il‐benchmar. All figures presented in this work were created using PyMOL^[83]^ (Figure 1), LaTeX/tikz (Figure 2) or matplotlib^[84]^ (Figures 3 to 6).

## Tutorial Part: Computational Strategies to Enhance the Reliability of Dynamic Properties

Although it is a commonly employed task, the calculation of transport properties using MD simulations can be a challenge, especially in highly viscous materials, such as ionic liquids.[^63^, ^85^, ^86^] As widely known by the scientific community, transport properties from MD simulations suffer from large statistical uncertainties that can be related to the limited sampling.[^63^, ^86^] Furthermore, the last step in the calculation of the transport properties may, depending on the quantity of interest, involve linear or nonlinear fitting. However, this can impose limitations on the reproducibility and accuracy of the calculated properties, for example, when determining the linear (diffusive) regime of the mean squared displacement. These challenges have been debated multiple times in the literature in recent years.[^63^, ^86^, ^87^]

To overcome the challenges of reproducibility and accuracy that are inherent in the calculation of transport properties of ILs using MD simulations, we developed computational strategies to obtain reliable quantities and statistical uncertainties. In this work, we address self‐diffusion coefficients, ionic conductivities, and local dynamics (rotational dynamics, and ion pair/ion cage lifetimes).

### Self‐Diffusion Coefficients

In MD simulations, the diffusion coefficient D can be calculated from the mean square displacement (MSD) of the particles,

(1) $$\mathrm{MSD}(\tau )={\left\langle {\left|r_{i}\left(t+\tau \right)-r_{i}\left(t\right)\right|}^{2}\right\rangle }_{t,i},$$

(2) $$D^{L}=\lim_{\tau \to \infty }\frac{1}{2n\tau }\mathrm{MSD}(\tau ),$$

where τ represents the correlation depth, i. e. the time lag used to calculate the shift in a particle's position, n corresponds to the dimensionality of the system (in our case, n=3 ) and $r_{i}$ is the time‐dependent position of the center of mass (or any other chosen reference point) of particle i . The parameter L denotes the edge length of the finite‐sized cubic simulation box and indicates that $D^{L}$ is the finite‐size diffusion coefficient. It is worth noting that, while Eq. 2 follows from the Einstein−Smoluchowski equation, the self‐diffusion coefficients are equally accessible through the Green−Kubo relation.[^88^, ^89^, ^90^]

Conventionally, D is obtained by linear regression of MSD vs. τ in the diffusive regime. However, since plots of MSD against τ commonly feature three main diffusion regimes (ballistic, sub‐diffusive, and diffusive), identifying the diffusive regime may not be a straightforward task. Moreover, at very large correlation depths, MSD(τ) can become noisy due to insufficient sampling. These observations highlight the need for a well‐defined and unbiased criterion to select a suitable diffusive regime to calculate the self‐diffusion coefficients from MSD(τ) . In TRAVIS, this is circumvented since, by default, a maximal correlation depth $\tau_{\max}$ of only 30 % of the total simulation time is used. As seen in Eq. 1, this does not mean that only 30 % of the trajectory is used, but that only displacements in this time regime are used for the calculation of $D^{L}$ . This is mathematically equivalent to calculating MSD(τ) for all possible τ and then discarding the last 70 % of the MSD for the linear fit, thus, no information is lost for the investigated range of τ values.

The evaluation of the β (τ ) parameter is a well established approach[^91^, ^92^] to identify the diffusive regime region in the logarithmic representation of MSD versus τ . Therefore, we can define β (τ ) as

(3) $$\beta \left(\tau \right)=\frac{\partial \ln\mathrm{MSD}(\tau )}{\partial \ln\tau }.$$

In the above expression, the diffusive regime is indicated by β (τ )≃ 1. We follow the recommendations of Humbert *et al*.^[93]^ to systematically scan the MSD(τ) data set and identify its diffusive regime based on the β (τ ) value. To do that, we implemented a Python routine, msdiff, available at https://github.com/kirchners‐manta/msdiff, that processes the MSD as a function of τ , as obtained from the TRAVIS output. We recommend following the TRAVIS default, using a maximal correlation depth $\tau_{\max}$ of 30 % of the simulation time (as also used in this work), leading to the MSD featuring only very little noise. Our routine, visualized in Figure 2 (left), starts from the output of an MSD calculation performed by TRAVIS (or any other software that calculates MSD (τ ) from a trajectory). Second, the data are initially divided into x slices of equal size. Third, x-1 extra slices are added, each of which is centered at the boundary between two neighboring slices from the initial partition. This process creates a total of 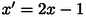 slices $S_{y}$ with 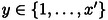 , where adjacent slices overlap, and all slices have the same width (see Figure 2, right). Then, for every slice (starting with 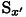 , i. e., at the largest correlation depths), the slope of the MSD in the doubly logarithmic plot of the respective slice is determined. If β(τ)≃1 within a tolerance δ , the slice is gradually expanded towards smaller correlation depths by adding more data points until $\beta \;\left(\tau \right)\notin \left[1-\delta ,1+\delta \right]$ is fulfilled. Otherwise, the slice is discarded and not considered further. After iterating over all slices, the linear regression is performed on the largest coherently linear interval of the MSD.

It is important to note that the identification of the linear regime is, besides the sampling of the MSD, mainly influenced by the aforementioned technical parameters. Empirically, we found 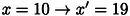 , δ=0.1 and the incremental addition of 1 % of the entire MSD data during the slice expansion to work well. By adhering to this systematic procedure, we could effectively identify distinct linear regimes for each data set. Given a sufficiently sampled trajectory, this routine ensures a reliable and robust calculation of the self‐diffusion coefficients, since it accounts for the behavior of the MSD and adapts the linear regression analysis accordingly. Beyond that, the small influence of the user bias ensures better reproducibility.

In Figure 3, we illustrate three arbitrary MSD data sets in a linear representation (top panel). Noise in data sets 1 and 2 (black and red lines, respectively) was created artificially to illustrate the effects of too large correlation depths during the MSD calculation. A sufficiently well‐sampled trajectory in conjunction with a maximal correlation depth $\tau_{\max}$ of around 30 % of the length of the production run would usually yield a relatively linear MSD data set 3 (blue). The bottom panel of Figure 3 features logarithmic representations of the three data sets along with their corresponding linear regimes that were identified by the procedure described above.

As is well known, the calculation of self‐diffusion coefficients from MD simulations can suffer from size effects.[^94^, ^95^, ^96^] To avoid this issue, we incorporate the additive correction term K proposed by Yeh and Hummer^[97]^ into our post‐processing routine. This correction term is derived from the Stokes−Einstein equation, and defined as

(4) $$K=\frac{k_{B}T\xi }{6\pi \eta L},$$

using the box edge length L , Boltzmann constant $k_{B}$ , temperature T , dynamic viscosity η and a dimensionless parameter ξ=2.837298 . The infinite box size diffusion coefficient $D^{\infty }$ is then calculated from the finite‐size diffusion coefficient $D^{L}$ as

(5) $$D^{\infty }=D^{L}+K=\frac{1}{6}\frac{\partial \mathrm{MSD}(\tau )}{\partial \tau }+\frac{k_{B}T\xi }{6\pi \eta L}.$$

The present work investigates diffusion in ionic liquids and, therefore, the self‐diffusion coefficients of anions and cations are denoted by $D_{-}$ and $D_{+}$ , respectively.

### Ionic Conductivity

Similarly to the self‐diffusion coefficients, the ionic conductivity, σ , can be calculated on the basis of MD simulations using two distinct approaches: the Green−Kubo method, which relies on the collective current of the particles, and the Einstein−Helfand method, based on the collective mean square displacement (MSD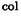 ) of the particles.^[98]^ However, unlike diffusivity, the ionic conductivity is a collective property, thus imposing even more difficulties in its calculation based on MD simulations and requiring better sampling.[^87^, ^98^]

In this work, we focus on the Einstein−Helfand approach and use a similar notation as Schröder *et al*.^[98]^ Therein, $\sigma_{\mathrm{EH}}$ , denoting the Einstein−Helfand (EH) conductivity, obeys the following expression:

(6) $$\sigma_{\mathrm{EH}}=\frac{e^{2}}{6Vk_{B}T}\lim_{\tau \to \infty }\frac{\partial }{\partial \tau }{\left\langle \sum_{i,j}^{N}q_{i}q_{j}\Delta r_{i}\left(t,\tau \right)\Delta r_{j}\left(t,\tau \right)\right\rangle }_{t},$$

using the elementary charge e , the simulation box volume V , the Boltzmann constant $k_{B}$ , and temperature T . In addition, N denotes the number of particles (ions), and $q_{i},q_{j}$ are the charges of the particles i and j , respectively. Finally, we define 7

(7) $$\Delta r_{i}\left(t,\tau \right)=r_{i}\left(t+\tau \right)-r_{i}\left(t\right),$$

where, as for the MSD, $r_{i}$ is the time‐dependent position vector of the center of mass (or any other chosen reference point) of ion i .

Since ionic conductivity is a collective property, it can be partitioned into its partial contributions from specific ionic interactions in the systems, i. e., cation‐cation, anion‐anion and cation‐anion interactions.^[99]^ These can be further decomposed into self‐contributions $\sigma_{z}^{\mathrm{self}}$ and cross‐contributions $\sigma_{\mathrm{zz}}^{\mathrm{cross}}$ and $\sigma_{+-}^{\mathrm{cross}}$ , where z refers to the sign of the charge of the involved species, that is, + or - . The interested reader is kindly referred to Section 3 of the Supporting Information for a comprehensive overview of the equations of all contributions to $\sigma_{\mathrm{EH}}$ . Eq. 6 be written as

(8) $$\sigma_{\mathrm{EH}}=\sigma_{+}^{\mathrm{self}}+\sigma_{-}^{\mathrm{self}}+\sigma_{++}^{\mathrm{cross}}+\sigma_{--}^{\mathrm{cross}}+\sigma_{+-}^{\mathrm{cross}}.$$

In systems with only two types of ions, the self terms $\sigma_{z}^{\mathrm{self}}$ can also directly be accessed from the self diffusion coefficients via:

(9) $$\sigma_{z}^{\mathrm{self}}=\frac{e^{2}}{Vk_{B}T}\cdot N_{z}q_{z}^{2}D_{z}.$$

Consequently, the self ionic conductivity (also known as Nernst−Einstein ionic conductivity) $\sigma_{\mathrm{NE}}$ is straightforwardly accessible through the calculation of the self diffusion coefficients and is defined as^[100]^

(10) $$\sigma_{\mathrm{NE}}=\sigma_{+}^{\mathrm{self}}+\sigma_{-}^{\mathrm{self}}.$$

Despite the fact that cross‐correlations are neglected in Eq. 10, this quantity provides valuable information on charge transport in the system under investigation. In particular, $\sigma_{\mathrm{EH}}$ simplifies to $\sigma_{\mathrm{NE}}$ in the limit of infinite dilution, and hence can be understood as “ideal ionic conductivity”. However, in highly concentrated ionic systems, such as ionic liquids, cross‐correlation terms cannot be neglected without significantly influencing the result.^[86]^

Although $\sigma_{\mathrm{NE}}$ has several limitations, it is still a very useful approach to investigate how the ionic interactions affect the charge transport or to evaluate the ionicity in the system, which can be done using the inverse Haven ratio $H_{R}^{-1}$ approach, defined as^[101]^

(11) $$H_{R}^{-1}=\frac{\sigma_{\mathrm{EH}}}{\sigma_{\mathrm{NE}}},$$

However, we would like to highlight that the validity and applicability of $H_{R}^{-1}$ for ionic liquids and molten salt systems has debated in the last years.^[102]^

Furthermore, insights on the contribution of each ionic species to the overall charge transport can be gained from the transport numbers, or transference numbers, $t_{z}$ . It is important to distinguish between the ideal transference numbers $t_{z}^{\mathrm{id}}$ and real transference numbers $t_{z}^{r}$ , defined as

(12) $$t_{z}^{\mathrm{id}}=\frac{\sigma_{z}^{\mathrm{self}}}{\sigma_{\mathrm{NE}}},$$

(13) $$t_{z}^{r}=\frac{\sigma_{z}^{\mathrm{self}}+\sigma_{\mathrm{zz}}^{\mathrm{cross}}+\frac{1}{2}\sigma_{+-}^{\mathrm{cross}}}{\sigma_{\mathrm{EH}}}.$$

In the limit of infinite dilution, Eq. 13 will simplify to Eq. 12. As already pointed out by Shao *et al*.^[103]^ and Harris *et al*.,^[104]^ $t_{z}^{r}$ depends highly on the choice of the reference frame in binary systems and thus will not be discussed here. However, other dynamic properties, namely $D_{z}$ and $\sigma_{\mathrm{EH}}$ (and consequently, $\sigma_{\mathrm{NE}}$ and $t_{z}^{\mathrm{id}}$ ) are independent of the chosen reference frame.

Similarly to the self‐diffusion coefficients, the conductivities $\sigma_{\mathrm{EH}}$ and their contributions are also obtained from the linear regression of the collective MSD. We apply the same post‐processing routine as for the calculation of self‐diffusion coefficients (see Figure 2) and identify the linear regime by γ(τ)≃1 , where

(14) $$\gamma \left(\tau \right)=\frac{\partial \ln{\mathrm{MSD}}^{\mathrm{col}}\left(\tau \right)}{\partial \ln\tau }.$$

Unlike the non‐collective MSD, that usually exhibits the linear regime from medium to long correlation depths, there is often no such clear tendency found in the collective MSD, due to insufficient sampling.^[98]^ For a given trajectory, the collective MSD is often much more noisy than the non‐collective MSD, making the search for its linear region more difficult. As can be seen in Figure 4 and observed in other studies,[^86^, ^87^, ^98^] the cross terms can show nonlinear behavior (violet and yellow curves), highlighting the demand for a sophisticated approach to obtain reliable conductivities, especially if the cross and self contributions are of interest. To ensure the comparability of the different contributions to the conductivity obtained from a specific trajectory, we choose to perform the linear regressions for all individual self and cross terms in the same linear regime region, determined from the collective MSD (including all self and cross terms). For [C2C1Im][NTf2] this procedure seems suitable, however, it may not be suitable for ionic liquids or other systems in which the cation and anion present very different mobilities.

### Local Dynamics

The calculation of an arbitrary life time 𝒯 is based on a corresponding correlation function C(τ) , with τ again being the correlation depth. In the case of rotational (or reorientation) dynamics, C is the autocorrelation of a vector along which the reorientation is measured. For the calculation of the ion pair and ion cage lifetimes, geometric criteria are used to define an ion pair or ion cage, and the autocorrelation of an auxiliary quantity indicating whether these criteria are met is computed.

We define an ion pair as an ion and its next‐neighbor counterion, whereas ion cages are formed by a central ion and all its counterions within the first solvation shell obtained from the location of the first minimum of the corresponding radial distribution function, g(r) . It should be noted that ion pair/cage lifetimes can be calculated in an intermittent (i. e., breaking and reforming of pair/cage condition is allowed) or in a continuous fashion (i. e., breaking and reforming of aggregates is *not* allowed). For a more comprehensive overview of this matter, we refer the reader to our previous works.[^80^, ^105^]

In general, 𝒯 is obtained straightforwardly from the integral of the autocorrelation, multiplied by a constant a :

(15) $$𝒯=a\int_{0}^{\infty }C\left(\tau \right)d\tau .$$

Note that a=1 for the reorientation time and a=2 for the ion pair/ion cage time. Due to the slow dynamics of the IL systems, it is common that C does not fully converge to zero within the simulation time. Thus, it is common practice to fit an exponential function of the form

(16) $$f\left(\tau \right)=\sum_{i}c_{i}\exp\left(-\frac{\tau }{b_{i}}\right)$$

to the autocorrelation. In this expression, i is the number of terms in the above sum, and $c_{i},b_{i}$ are the fitting parameters of the respective term. The integral can be solved analytically $\forall c_{i}\geq 0\wedge b_{i}>0$ and by inserting the result into Eq. 15, the life time is computed as,

(17) $$𝒯=a\sum_{i}c_{i}b_{i}.$$

Fitting an autocorrelation becomes more involved, the more terms are included in the expansion (Eq. 16), because the number of fit parameters increases. Therefore, constraints on the parameter space are often invaluable in solving this problem. In addition to the requirements mentioned above for $b_{i}$ and $c_{i}$ , the sum of all $c_{i}$ can be required to be 1 if C(τ=0)=1 .

It is worth noting that throughout this work, an exponential fit function with two terms was empirically found to represent the data well and thus, Eq. 16 can be explicitly written as

(18) $$f_{2}\left(\tau \right)=c_{1}\exp\left(-\frac{\tau }{b_{1}}\right)+c_{2}\exp\left(-\frac{\tau }{b_{2}}\right).$$

The corresponding lifetime is obtained as

(19) $$𝒯=a(c_{1}b_{1}+c_{2}b_{2}).$$

### Uncertainty Quantification

Building on the established methodology to obtain self‐diffusion coefficients, ionic conductivities, and lifetimes, our focus is on estimating their uncertainties. As mentioned above, accurate dynamic properties from MD simulations heavily rely on sufficient sampling. To this end, conducting independent replica simulations is beneficial and, in most cases, allows for efficient parallelization (compared to conducting a single, very long simulation). With several replica trajectories at hand, there are two popular approaches: In the first approach, observables (i. e., MSD, collective MSD, autocorrelation) are calculated and fits are performed *individually* for every trajectory. Then, overall properties (D,σ,𝒯 ) are obtained as the arithmetic mean of the individual properties obtained from the replica trajectories, while the standard deviation can be interpreted as the uncertainty of the set of individual trajectories. It should be noted that the individual fits also carry uncertainties, but these are usually much smaller than the uncertainties from the trajectories and hence, neglected here.

In the second approach, observables are calculated for every trajectory individually and then averaged. Subsequently, the overall properties are obtained from fitting the *averaged* observables. Then, the inverse of the variance of each data point in the observable can be used as weight of the respective data point in an error weighted fit. The uncertainty of this approach is the uncertainty of the weighted fit, not comparable to and usually much smaller than the uncertainty calculated in the first approach. An example of both approaches and the resulting uncertainties of the diffusion coefficient is discussed in the Results section. Furthermore, in Section 4 of the Supporting Information, we provide an estimate of the maximum possible error introduced on the conductivities by averaging the observables before fitting.

The second approach is more robust than the first one, meaning that increasing the sampling by averaging the observables decreases noise in the data. Considering the present example of identifying a linear regime in the MSD, averaging facilitates larger linear regimes. We found that to be particularly important in view of the ionic conductivities, where for some individual trajectories (approach 1), no linear regime in the collective MSD could be identified. As a consequence, we use approach 2 for all dynamic properties in this work, that is, self‐diffusion coefficients, ionic conductivities, and lifetimes.

To further propagate uncertainties, we employ the well‐established Gaussian error propagation. The uncertainty Δf of a quantity $f(x_{1},x_{2},...,x_{n})$ is dependent on a set of variables $x_{i}$ and is defined as

(20) $$\Delta f=\sqrt{\sum_{i}{\left(\frac{\partial f}{\partial x_{i}}\Delta x_{i}\right)}^{2}}$$

with the individual uncertainties $\Delta x_{i}$ . Applying this to the Yeh−Hummer correction for finite size simulation boxes (Eq. 5) yields

(21) $$\Delta D^{\infty }=\sqrt{{\left(\Delta D^{L}\right)}^{2}+{\left(\Delta K\right)}^{2}}$$

and

(22) $$\Delta K=\frac{k_{B}T\xi }{6\pi \eta^{2}L}\Delta \eta .$$

The expression for K from Eq. 4 was initially derived for application to MD simulations, considering the simulation temperature and the box size as the “true” values. However, the viscosity is taken from experiments (or other calculations) and thus carries an uncertainty itself. The Gaussian error propagation is similarly employed to estimate the uncertainties of the transference numbers (Eq. 12

(23) $$\Delta t_{z}^{\mathrm{id}}=\sqrt{{\left(\frac{\Delta \sigma_{z}^{\mathrm{self}}}{\sigma_{\mathrm{NE}}}\right)}^{2}+{\left(\frac{\sigma_{z}^{\mathrm{self}}\Delta \sigma_{\mathrm{NE}}}{\sigma_{\mathrm{NE}}^{2}}\right)}^{2}}\;$$

and lifetimes (Eq. 19

(24) $$\Delta 𝒯=a\sqrt{{\left(b_{1}\Delta c_{1}\right)}^{2}+{\left(c_{1}\Delta b_{1}\right)}^{2}+{\left(b_{2}\Delta c_{2}\right)}^{2}+{\left(c_{2}\Delta b_{2}\right)}^{2}}.$$

Reference self‐diffusion coefficients $D^{\mathrm{ref}}$ are derived from experiments by Tokuda *et al*.^[106]^ In their manuscript, the authors provide a Vogel−Fulcher−Tamman equation to calculate the diffusion coefficients of several ILs at desired temperatures. The reference values are determined according to

(25) $$D^{\mathrm{ref}}=D_{0}\exp\left(-\frac{B}{T-T_{0}}\right)$$

with the parameters $D_{0},B,T_{0}$ and their uncertainties provided in the paper, and T=350k (the simulation temperature). The uncertainty of $D^{\mathrm{ref}}$ was calculated according to Eq. 20. Similarly, the reference viscosity and its uncertainty (required for Eqs. 4 and 22) were calculated from a Vogel−Fulcher−Tamman equation provided by Tokuda *et al.*.^[106]^

## Results and Discussion

In the following, we examine the self‐diffusion coefficients, ionic conductivity, and local dynamics obtained from classical MD simulations using different force fields.

### Self‐Diffusion Coefficients

Figure 5 shows the (finite box size corrected) self‐diffusion coefficients obtained from the MD simulations of the anion ${[\mathrm{NTf}}_{2}{]}^{-}$ , $D_{\infty }^{-}$ , in the bottom panel (red bars) and those of the cation [C2C1Im]+ , $D_{\infty }^{+}$ , (blue bars) in the center panel. The calculated self‐diffusion coefficients are compared to reference data from Ref. [106], specifically $D_{+}^{\mathrm{ref}}=(197.5\pm 46.5)$ $\cdot 10^{-12}m^{2}s^{-1}$ and $D_{-}^{\mathrm{ref}}=\left(132.1\pm 25.8\right)$ $\cdot 10^{-12}m^{2}s^{-1}$ .

It is observed that the cations exhibit faster diffusion than the anions. The self‐diffusion coefficients from the simulations using force fields with unity charges, i. e., MD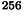 , MD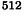 , and MD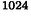 , are predicted as approximately $35\cdot 10^{-12}m^{2}s^{-1}$ and $21\cdot 10^{-12}m^{2}s^{-1}$ for cations and anions, respectively, and thus are systematically too small by a factor of around six. Furthermore, the self‐diffusion coefficients obtained from the polarizable force field simulations (polMD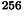 ), as well as the simulations with scaled charges (MD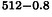 ) and refined LJ parameters (NGOLP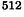 ) meet the reference within its uncertainty. Although polMD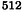 slightly overestimates the self‐diffusion coefficient of the anion, the prediction is still in the correct order of magnitude.

It is well‐known that integer charges result in slow dynamics in purely ionic systems, due to the strong, long‐ranged Coulomb interactions, and neglect important charge transfer phenomena and electronic polarization. Down‐scaling the charges or adding explicit polarization are popular approaches to accelerate the dynamics of ILs. Consequently, the aforementioned findings agree with previous studies.^[32]^ It is noteworthy that the NGOLP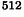 simulations, using refined LJ parameters for the anion, capture the reference data equally well as the polarizable and charge‐scaled MD simulations but without the increased computational cost of the former and the structural deficiencies of the latter.^[59]^

However, the purpose of this manuscript is not to benchmark existing methods and force fields against reference data, but rather to provide a perspective on the reliable calculation of IL transport properties and their uncertainties. Therefore, the top panel of Figure 5 displays the ratio of the calculated diffusion coefficients of cations and anions $\frac{D_{+}^{\infty }}{D_{-}^{\infty }}$ . It is evident that the ratios obtained from all systems are very close to the references and well‐captured within the reference uncertainty. That result is intriguing, showing that, despite the simulations with unity charges consistently feature too slow dynamics, their qualitative behavior (i. e., the relative diffusion of cations and anions with respect to each other) is displayed correctly. If this observation proved true for other dynamic properties as well, that could have a notable impact on future studies where quantitative agreement to experiments might not be necessary, but qualitative agreement is sufficient.

To comprehensively discuss the uncertainties in the calculated self‐diffusion coefficients, the values shown in Table 2 are examined. It should be noted that we sacrifice consistency in terms of significant digits here to display all values and their uncertainty with the same number of decimal places, thereby enabling comparison of the magnitudes of the uncertainties. As discussed in the Uncertainty Quantification section of the Tutorial Part, we calculate the finite size corrected self‐diffusion coefficient $D^{\infty }$ from the finite size self‐diffusion coefficient $D^{L}$ and the Yeh−Hummer correction^[97]^ K . We stress here, that we refer to $D^{L}$ as the finite size self‐diffusion coefficient calculated by approach 2 (linear regression on the averaged MSD of all simulations). For comparison and discussion, we also show $D^{L,\mathrm{ave}}$ , the finite size self‐diffusion coefficient obtained from approach 1 (linear regression of the MSD of every simulation and subsequent averaging).

**Table 2:** Finite box size diffusion coefficients $D^{L}$ and $D^{L,\mathrm{ave}}$ , Hummer correction terms K , infinite box size diffusion coefficients $D^{\infty }$ and their uncertainties $\Delta D^{L}$ , $\Delta D^{L,\mathrm{ave}}$ , ΔK , $\Delta D^{\infty }$ (all in $10^{-12}m^{2}s^{-1}$ ). The data is visualized in Figure 5.

Ion	System	$D^{L}$	$\Delta D^{L}$	$D^{L,\mathrm{ave}}$	$\Delta D^{L,\mathrm{ave}}$	K	ΔK	$D^{\infty }$	$\Delta D^{\infty }$
[C2C1Im]+	MD 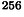	35.176	0.004	35.033	1.243	18.045	10.986	53.221	10.986
	MD 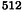	34.862	0.005	34.701	0.894	14.325	8.721	49.187	8.721
	MD 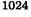	35.861	0.001	35.779	0.393	11.372	6.923	47.233	6.923
	MD 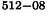	152.005	0.011	151.497	1.088	14.180	8.632	166.184	8.632
	NGOLP 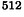	221.801	0.028	219.630	7.211	14.347	8.735	236.149	8.735
	polMD 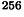	192.438	0.013	193.267	5.662	17.963	10.936	210.402	10.936
	polMD 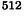	214.769	0.036	212.730	3.319	14.180	8.633	228.949	8.633
	Ref. [106]	–	–	–	–	–	–	197.541	46.466
${\left[{\mathrm{NTf}}_{2}\right]}^{-}$	MD 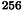	20.567	0.003	20.678	0.815	18.045	10.986	38.612	10.986
	MD 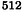	20.873	0.002	20.794	1.033	14.325	8.721	35.199	8.721
	MD 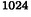	21.016	0.003	20.894	0.371	11.372	6.923	32.388	6.923
	MD 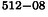	102.778	0.005	102.630	1.033	14.180	8.632	116.958	8.632
	NGOLP 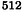	127.863	0.012	127.902	1.705	14.347	8.735	142.211	8.735
	polMD 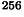	139.851	0.019	140.189	3.745	17.963	10.936	157.815	10.936
	polMD 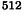	156.813	0.039	153.773	5.771	14.180	8.633	170.993	8.633
	Ref. [106]	–	–	–	–	–	–	132.066	25.771

Firstly, $D^{L}$ is always found close to $D^{L,\mathrm{ave}}$ and within its uncertainty $\Delta D^{L,\mathrm{ave}}$ , which is the standard deviation of the individual simulations. Secondly, the relative uncertainty of $D^{L,\mathrm{ave}}$ is always below 5 %. From that, we conclude, that the individual simulations of each system do not exhibit large spreads of their MSDs among each other. The uncertainty $\Delta D^{L}$ (originating from the error weighted fit) is very small and almost negligible, indicating the high linearity of the regime in which the fit is performed. In the present case $\Delta D^{L}$ is about 2 to 3 orders of magnitude smaller than $\Delta D^{L,\mathrm{ave}}$ but it should be mentioned that these two uncertainties originate from distinctly different sources, thus describing different kinds of uncertainties and are not interchangeable, which is discussed in the Tutorial Part in more detail.

To extrapolate $D^{L}$ to an infinite simulation box size, the correction term K is added. Notably, the correction is inversely proportional to the size of the box and thus is particularly relevant to small systems (see 7th column of Table 2) Moreover, when viscous liquids with slow dynamics, such as ILs, are investigated, the correction often constitutes a significant part of $D^{\infty }$ . For example, it constitutes >45% to $D^{\infty }$ of the anion for MD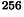 system and still contributes 11 % to the polMD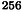 diffusion coefficient. We note here that the Yeh−Hummer correction was initially developed for non‐ionic systems with faster dynamics, but to the best of our knowledge, there is no clear evidence in the literature that this correction is not applicable to ILs.

Additionally, the uncertainty ΔK , obtained from the Gaussian error propagation, is relatively large compared to the actual value of K . Its magnitude originates from the uncertainty of the reference viscosity.^[106]^ As a consequence, ΔK is also considerably larger than $\Delta D^{L}$ in most cases, resulting in $\Delta D^{\infty }$ being approximately the same as ΔK . This results in large relative uncertainties in the diffusion coefficients of systems with slow dynamics (simulations with unity charges).

### Ionic Conductivity

The ionic conductivities calculated using the Nernst−Einstein ($\sigma_{\mathrm{NE}}$ ) and Einstein−Helfand ($\sigma_{\mathrm{EH}}$ ) formalisms are depicted in the bottom panel of Figure 6 as orange and purple bars, respectively. Several experimentally derived reference values of the ionic conductivity, including uncertainties, from Refs. [106, 107, 108, 109, 110, 111, 112], were considered, and are listed in detail in Section 5 of the Supporting Information. In Figure 6, we show the weighted average of these values as a black dashed line and its uncertainty in gray, given as $\sigma^{\mathrm{ref}}=\left(3.01\pm 0.11\right){Sm}^{-1}$ . We emphasize again that, due to its statistical sensitivity, it was not possible to identify a linear regime in the collective MSD of each individual simulation. To this end, as described in the Tutorial Part, we calculated the ionic conductivities by a linear regression of the averaged collective MSD of each system.

It is evident, that for all systems the Nernst−Einstein conductivity $\sigma_{\mathrm{NE}}$ is larger than the Einstein−Helfand conductivities $\sigma_{\mathrm{EH}}$ . This is a consequence of the sum of the cross terms being consistently smaller than zero, thus reducing the overall conductivity, and showing strong ionic correlation effects. The source of this effect is not straightforwardly interpretable because the magnitudes of the individual cross terms strongly depend on the chosen frame of reference. The influence of the cross terms on the ionic conductivity is further quantified by the ionicity (see Eq. 11), visualized in the last column of Table 3. For the systems studied, the ionicity ranges from 0.68 (polMD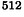 ) to 0.92 (MD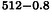 ) but lacks a solid trend. The difference in the ionicity between the polMD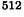 and polMD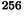 systems (0.68 vs. 0.91) is probably related to the spread of the collective MSD of the individual simulations, which is discussed in more detail in Section 4 of the Supporting Information. That points to the suggestion that five independent trajectories (as performed in this work) might not be enough to fully converge the Einstein−Helfand ionic conductivity in simulations using polarizable force fields.

**Table 3:** Ionic conductivities calculated using the Nernst−Einstein ($\sigma_{\mathrm{NE}}$ ) and Einstein−Helfand ($\sigma_{\mathrm{EH}}$ ) formalisms and their uncertainties in comparison to experimentally derived reference data (all in $S\;\;m^{-1}$ ) as well as the corresponding ionicities (dimensionless). Visualization of the data is shown in Figure 6.

System	$\sigma_{\mathrm{NE}}$	$\Delta \sigma_{\mathrm{NE}}$	$\sigma_{\mathrm{EH}}$	$\Delta \sigma_{\mathrm{EH}}$	$\frac{\sigma_{\mathrm{EH}}}{\sigma_{\mathrm{NE}}}$
MD 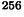	0.6669	0.0002	0.5832	0.0002	0.88
MD 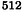	0.6680	0.0002	0.5618	0.0002	0.84
MD 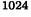	0.6766	0.0001	0.6051	0.0001	0.89
MD 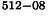	2.8908	0.0001	2.6626	0.0004	0.92
NGOLP 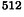	4.1785	0.0001	3.2875	0.0001	0.79
polMD 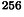	3.8751	0.0001	3.5217	0.0005	0.91
polMD 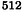	4.3049	0.0005	2.2940	0.0009	0.68
Ref.	–	–	3.0057	0.1119	–

To compare the calculated ionic conductivities to the reference data quantitatively, Table 3 is examined. The results from the MD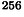 , MD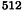 and MD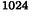 simulations describe the ionic conductivity poorly and yield values that are systematically too small by a factor of around five to six. Unlike that, the other systems approach the reference values much better. The calculated Nernst−Einstein ionic conductivities for NGOLP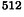 , polMD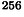 and polMD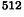 overestimate the references, while the prediction for MD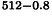 is at the lower bound of the reference. For NGOLP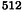 and the polarizable systems, the Einstein−Helfand ionic conductivities are closer to the references than the Nernst−Einstein conductivities and yield a very good agreement. It is remarkable that quantitatively correct ionic conductivities can even be obtained using a nonpolarizable force field, precisely by refining the LJ parameters as done for the NGOLP force field. Other than that, we emphasize that considering the cross terms of the collective MSD is crucial when aiming at quantitative ionic conductivities. Lastly, it should be mentioned that the uncertainties of $\sigma_{\mathrm{NE}}$ and $\sigma_{\mathrm{EH}}$ , obtained from the weighted fit of the collective MSD, are very small and negligible in this case.

To investigate the qualitative behavior of the ionic conductivity of different force fields, the ideal transport numbers $t_{z}^{\mathrm{id}}$ , displayed in the top panel of Figure 6, are often used. However, upon considering Eqs. 9 and 13, $t_{z}^{\mathrm{id}}$ effectively reduces to $\frac{D_{z}}{D_{+}+D_{-}}$ , and hence, contains the same information as the top panel of Figure 5. It can be concluded that all systems show a very similar qualitative behavior when comparing the contributions of cations and anions to the Nernst−Einstein conductivity (i. e., the charge transport in the systems). As already discussed in the Tutorial Part, there is no analogous comparison for the Einstein−Helfand conductivity because calculated real transference numbers from MD simulations of binary ionic systems (in the barycentric frame of reference) are solely dependent on the mass ratio of the ions, while for other frames of reference, there are similar issues.[^103^, ^104^]

### Lifetimes and Reorientation Dynamics

Having discussed the global movement of molecules in the IL simulations, we now shift our focus to the investigation of local dynamics, namely ion pair/ion cage lifetimes and reorientation times (also known as rotational relaxation times) of the cations.

Reorientation dynamics are particularly interesting quantities to calculate from MD simulations, as they can be related to certain nuclear magnetic resonance (NMR) and electron paramagnetic resonance (EPR) spectroscopy experiments. To investigate the rotation of the cation, three vectors perpendicular to each other are defined (using the atom labels from Figure 1): $\vec{a}$ connects C2 and the adjacent hydrogen atom, while $\vec{b}$ connects the two nitrogen atoms N1 and N2, and $\vec{c}$ is standing perpendicular to the plane generated by C2, C3 and C4 (that is, the ring plane).

The calculated reorientation times 𝒯 and their uncertainties Δ𝒯 are displayed in Table 4. It is important to note that the 512 IP systems are shown exclusively, as these systems feature all relevant trends. The full table, including all seven systems, is provided in Section 6 of the Supporting Information.

**Table 4:** Reorientation times 𝒯 of the cations (in ps) for all 512 ion pair systems. Three different vectors, oriented perpendicular to each other (cf. Figure 1), were considered in the reorientation analyzes: $\vec{a}$ connects C2 and the adjacent hydrogen atom, $\vec{b}$ connects the two nitrogen atoms N1 and N2, and $\vec{c}$ stands perpendicular to the plane created by C2, C3 and C4 (i. e., the ring plane).

	${\vec{a}}_{C2,H}$		${\vec{b}}_{N1,N2}$		${\vec{c}}_{C2,C3,C4}$	
System	𝒯	Δ𝒯	𝒯	Δ𝒯	𝒯	Δ𝒯
MD 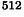	72.2	0.5	318.2	1.1	84.8	0.4
MD 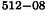	24.9	0.1	97.7	0.1	30.4	0.1
NGOLP 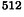	28.0	0.1	102.4	0.1	27.6	0.1
polMD 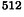	24.6	0.1	73.8	0.1	28.9	0.1

First, a clear trend in relaxation times is observed, independent of the system: ${𝒯}_{\vec{a}}\leq {𝒯}_{\vec{c}}{\ll 𝒯}_{\vec{b}}$ . This trend can be rationalized by recalling that the reorientation time of a molecule along a vector is proportional to the spatial extension of the molecule along this vector. For example, the extension of the cation along the N−N axis is the largest, due to the methyl and ethyl groups attached to the nitrogen atoms, and consequently, the vector ${𝒯}_{\vec{b}}$ has the slowest relaxation time, while ${𝒯}_{\vec{a}}$ presents the fastest relaxation.

When comparing the different systems, it is observed that the MD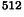 dynamics are roughly three times slower than the dynamics observed in the other three systems. The latter systems all exhibit reorientation times of similar magnitude. Consistently, the uncertainties of the reorientation times, originating from an error weighted fit, are very small.

To validate the calculated relaxation times, we compared the values for $\vec{a}$ with the experimental data from Wulf *et al*.^[113]^ They employed ^2^H magnetic relaxation experiments to study the rotational relaxation along the C2‐H vector and found a relaxation time of 13.1 ps at 343 K (see Tab. 1 of Ref. [113]). The calculated ${𝒯}_{\vec{a}}$ values are about a factor of two times larger than the experiment when considering the MD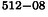 , NGOLP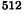 , and polMD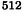 systems. As the quantitative prediction of relaxation times and lifetimes is a challenging task, the calculated results are deemed acceptable. Additionally, other works[^113^, ^114^, ^115^, ^116^, ^117^, ^118^] investigating the rotational dynamics of different ILs indicate that our calculated reorientation times are in the correct order of magnitude and are in line with previous findings. In contrast to that, the MD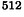 reorientation dynamics are found to be too slow.

Next, we turn to the discussion of ion pair and ion cage lifetimes. At this point, it is worth noting that the formation of ion pairs and ion cages, as well as their impact on the dynamics, is a long‐standing topic in the IL community.[^41^, ^119^, ^120^, ^121^, ^122^, ^123^, ^124^, ^125^, ^126^] The results of our calculations are presented in Table 5, but to keep this section brief and informative, we discuss the 512 ion pair simulations and continuous lifetimes only. Full information on all systems, including intermittent lifetimes, is available in Section 6 of the Supporting Information. A fair comparison to experimental data is not possible at this point because the ion pair and ion cage lifetimes cannot be measured directly.

**Table 5:** Ion pair (IP) and ion cage (CG) lifetimes 𝒯 of ${\left[{\mathrm{NTf}}_{2}\right]}^{-}$ around [C2C1Im]+ and their corresponding uncertainties (all in ps), for all 512 ion pair systems. Correlation functions were calculated in continuous fashion.

System	${𝒯}^{\mathrm{IP}}$	$\Delta {𝒯}^{\mathrm{IP}}$	${𝒯}^{\mathrm{CG}}$	$\Delta {𝒯}^{\mathrm{CG}}$
MD 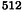	20.4	0.5	1253.2	3.5
MD 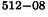	10.8	0.1	375.5	0.8
NGOLP 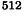	8.5	0.5	301.3	0.7
polMD 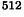	9.6	0.1	289.9	0.4

The lifetimes of the ion pairs are considerably shorter (more than one order of magnitude) than those of the ion cages (${𝒯}^{\mathrm{IP}}{\ll 𝒯}^{\mathrm{CG}}$ ). That is because the complete decomposition of an ion cage requires all ions within the first solvation shell to be removed from the vicinity of the central ion of the cage, whereas the decomposition of an ion pair solely requires two distinct ions to separate. We observe that the lifetimes of the MD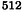 simulations are significantly larger than those obtained from the MD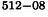 , NGOLP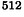 and polMD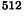 simulations. In addition, we note that the uncertainties of lifetimes from the error weighted fits are found to be very small.

In summary, it is remarkable that the rotational dynamics and ion pair/ion cage lifetimes of the MD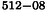 , NGOLP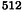 simulations are comparable to those using polarizable force fields, as was already observed for the diffusion coefficients and ionic conductivities.

## Conclusions

We presented a detailed tutorial on how to obtain reliable transport properties for ionic liquids from molecular dynamics simulations using http://www.travis‐analyzer.de/TRAVIS (our open‐source and freely available software) in conjunction with a custom Python routine available at https://github.com/kirchners‐manta/msdiff. In the tutorial, we discuss common pitfalls and put special emphasis on the calculation and discussion of uncertainties. This work features a new TRAVIS module for the calculation of ionic conductivities based on the collective mean squared displacement that will be available to the public in the near future. To apply the tutorial, molecular dynamics simulations of the ionic liquid [C2C1Im][NTf2] , employing various force fields, were presented, and their performance in terms of transport properties was discussed, including a comparison to reference data where possible. In particular, non‐polarizable force fields with unity charges and scaled charges, polarizable force fields with Drude oscillators, and non‐polarizable force fields with refined Lennard‐Jones parameters were considered in calculating self‐diffusion coefficients, ionic conductivities, rotational dynamics, and ion pair/ion cage lifetimes.

From our calculations, we observed that the simulations using the non‐polarizable CL&P force field with unity charges consistently feature too slow dynamics, which is in agreement with various previous studies. Scaling the molecular charges by an empirical factor of 0.8 accelerates the dynamics and leads to a reasonable agreement between the simulated and reference data (but is known to adversely affect structural properties that were not investigated in this work). The addition of explicit polarizability in the CL&Pol force field decreases the sluggishness of the dynamics, thus yielding very good agreement of the theoretical self‐diffusion coefficients and ionic conductivities with the reference data. Although the NGOLP force field also employs a unity charge model, transport properties using this force field are predicted very well and close to reference values, highlighting how reparametrization of the Lennard‐Jones parameters can be useful in improving the ILs’ transport properties’ description.

Another central point of this work was the investigation of the qualitative performance of the different force fields. To this end, the ratios of the calculated anion and cation self‐diffusion coefficients and ideal transference numbers were compared among all force fields. We found a good qualitative performance of all force fields considered in this work, which also translated to the rotational dynamics and lifetimes.

Our study highlights that all force fields can be employed to provide reliable physicochemical information from a qualitative point of view. However, the use of polarizable force fields or refinement of Lennard‐Jones parameters is required to improve the quantitative agreement with experimental reference data. The latter option is also very appealing from a resource point of view due to the lower computational costs compared to performing simulations with polarizable force fields. Although scaling the charges accelerates the dynamics, this approach tends to negatively affect the density and other structural features, and an additional reparametrization of other force field parameters may be necessary.

## Conflict of Interests

The authors declare no conflict of interest.

1

## Supporting information

As a service to our authors and readers, this journal provides supporting information supplied by the authors. Such materials are peer reviewed and may be re‐organized for online delivery, but are not copy‐edited or typeset. Technical support issues arising from supporting information (other than missing files) should be addressed to the authors.

Supporting Information

Supporting Information

Supporting Information

Supporting Information

Supporting Information

## Data Availability

The data that support the findings of this study are available in the supplementary material of this article.
