# Supplementary figures and images for: Lessons Learned on Obtaining Reliable Dynamic Properties for Ionic Liquids
Source: Chemphyschem. 2025 Feb 18;26(8):e202401048. doi: 10.1002/cphc.202401048 (PMC12005134; doi:10.1002/cphc.202401048)

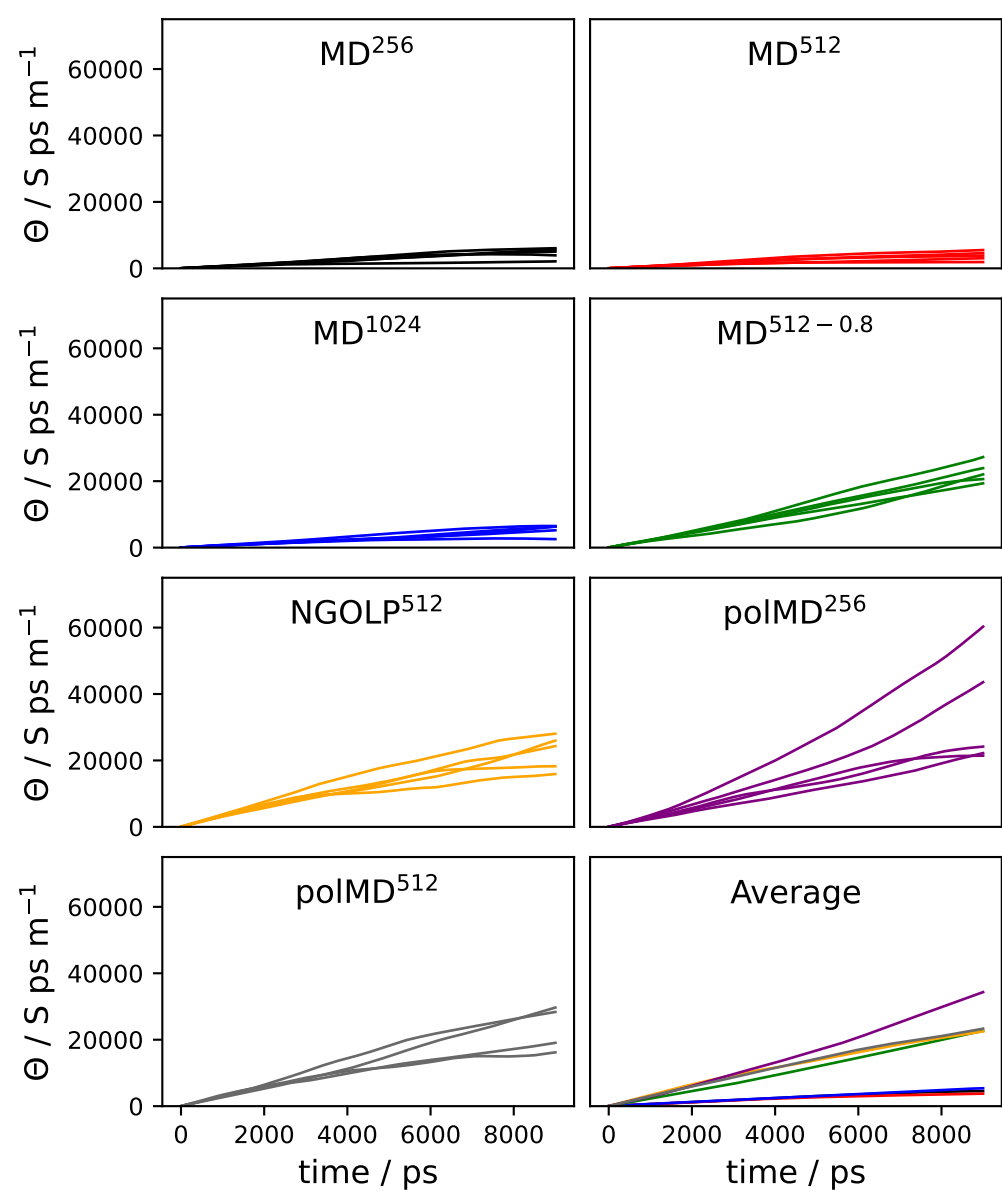

Supplement: Supplementary file 1 — Supporting Information [file CPHC-26-e202401048-s003.pdf]

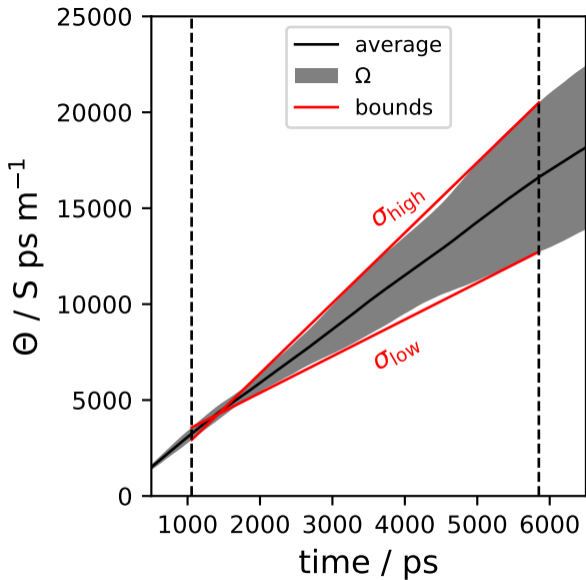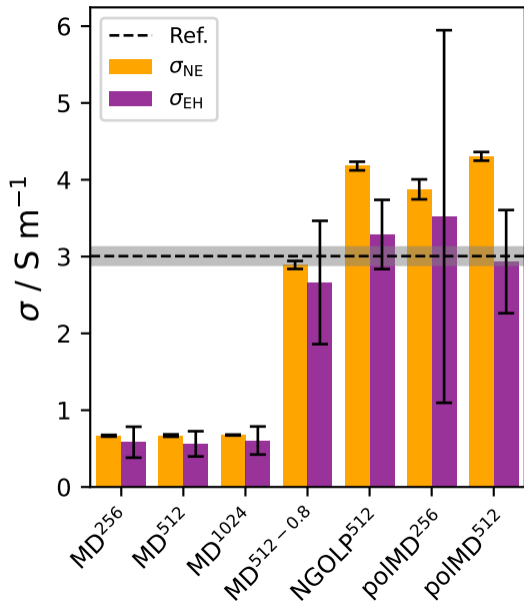

Supplement: Supplementary file 2 — Supporting Information [file CPHC-26-e202401048-s001.pdf]

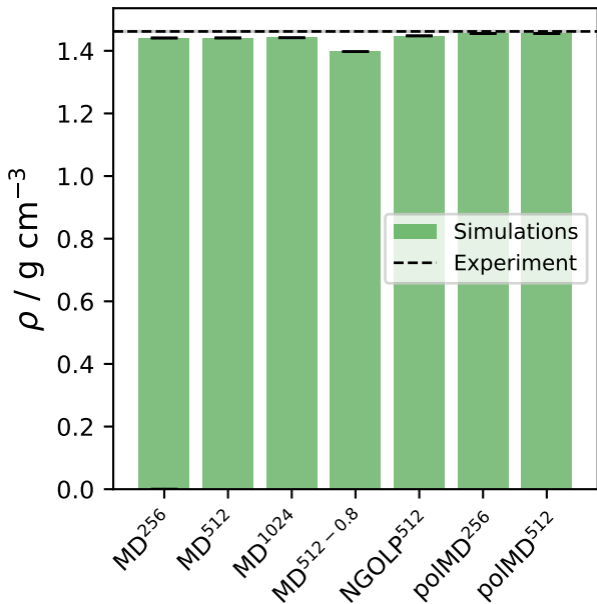

Supplement: Supplementary file 3 — Supporting Information [file CPHC-26-e202401048-s005.pdf]
