# Supplementary material for: Lessons Learned on Obtaining Reliable Dynamic Properties for Ionic Liquids
Source: Chemphyschem. 2025 Feb 18;26(8):e202401048. doi: 10.1002/cphc.202401048 (PMC12005134; doi:10.1002/cphc.202401048)
Supplement: Supplementary file 4 — Supporting Information [file CPHC-26-e202401048-s004.pdf]

ChemPhysChem

Supporting Information

Lessons Learned on Obtaining Reliable Dynamic Properties for Ionic Liquids

Tom Frömbgen, Paul Zaby, Vahideh Alizadeh, Juarez L. F. Da Silva, Barbara Kirchner,* and Tuanan C. Lourenço*

Supporting Information: Lessons learned on obtaining reliable dynamic properties for ionic liquids

Tom Frömbgen,† Paul Zaby,† Vahideh Alizadeh,† Juarez L. F. Da Silva,‡ Barbara Kirchner,*,† and Tuanan C. Lourenço*,†,‡

†*Mulliken Center for Theoretical Chemistry, University of Bonn, Beringstraße 4-6, D-53115 Bonn, Germany*

‡*São Carlos Institute of Chemistry, University of São Paulo, P.O. Box* 780, 13560-970, *São Carlos, SP, Brazil*

E-mail: kirchner@thch.uni-bonn.de; tuanan@usp.br

Contents

1 Force field parameters used in this study **S-2**

2 Densities of simulation boxes **S-4**

3 Conductivity equations **S-5**

4 Estimating the uncertainty of the conductivity **S-6**

5 Calculated and reference conductivities **S-10**

6 Local dynamics **S-11**

References **S-11**

1 Force field parameters used in this study

As stated in the main text, we used the CL&P[S1–S6] and the NGOLP[S7] force fields with their default parameters to perform all MD simulations. In the case of the CL&P three charge methods were used, i) unitary charges, ii) charges scaled by 0.8, and iii) the polarizable force field CL&Pol.[S8,S9]

Tables S1 and S2 show the atomic charges and Lennard-Jones parameters of the atom types found in $[C_2C_1Im]^+$ and $[NTf_2]^-$ and used in the simulations. Note that, except for the scaled charges, these are the default values of the respective force fields, i.e., no additional changes were made. Furthermore, as reported in the seminal works on the CL&Pol force field, the addition of the Drude particles leads to an overestimation of the Lennard-Jones cross interactions, i.e., the σ_{ij} and ϵ_{ij} obtained by the combination rules. Therefore, following the recommendations, all the ϵ_{ij} for the cation-anion interactions were scaled by the k_{ij}^{pred} term reported in Table S3, while the σ_{ij} were kept as default values. For a more comprehensive overview, the reader is referred to the work of Goloviznina et al.[S8]

Table S1: Atomic charges (in e) used in the MD simulations for all force fields. Note that the charges of CL&P^{Unitary} and CL&Pol are identical.

Atom	CL&P^{Unitary}	C&Pol$^{0.8}$	CL&Pol	NGOLP
NA	0.150	0.120	0.150	0.150
CR	−0.110	−0.088	−0.110	−0.110
CW	−0.130	−0.104	−0.130	−0.130
C1	−0.170	−0.136	−0.170	−0.170
HCR	0.210	0.168	0.210	0.210
C1A	−0.170	−0.136	−0.170	−0.170
HCW	0.210	0.168	0.210	0.210
H1	0.130	0.104	0.130	0.130
CE	−0.050	−0.040	−0.050	−0.050
HC	0.060	0.048	0.060	0.060
CBT	0.350	0.280	0.350	0.350
F1	−0.160	−0.128	−0.160	−0.160
SBT	1.020	0.816	1.020	1.020
NBT	−0.660	−0.528	−0.660	−0.660
OBT	−0.530	−0.424	−0.530	−0.530

Table S2: Lennard-Jones parameters σ and ϵ (in Å and $\mathrm{kJ\,mol^{-1}}$) used in the MD simulations for all force fields. Note that the CL&P parameters represents both unitary and scaled charges.

	CL&P		NGOLP	
Atom	σ	ϵ	σ	ϵ
NA	3.250	0.71128	3.2500	0.71128
CR	3.550	0.29288	3.0175	0.20501
CW	3.550	0.29288	3.5500	0.29288
C1	3.500	0.27614	3.5000	0.27614
HCR	2.420	0.12552	1.4520	0.18828
C1A	3.500	0.27614	3.5000	0.27614
HCW	2.420	0.12552	2.0570	0.08790
H1	2.500	0.12552	2.5000	0.12552
CE	3.500	0.27614	3.5000	0.27614
HC	2.500	0.12552	2.5000	0.12552
CBT	3.500	0.27614	3.1500	0.08282
F1	3.118	0.25540	2.6550	0.06651
SBT	3.550	1.04600	4.0825	0.31372
NBT	3.250	0.71128	3.2500	0.21333
OBT	3.150	0.83736	3.4632	0.26353

Table S3: Coefficients for scaling the ϵ_{ij} cross-interactions. r_{com} (in Å)is the center of mass distance between the ionic fragments, k_{ij}^{sapt} is the default scaling coefficient from SAPT calculations by Goloviznina et al.,[S8] and k_{ij}^{pred} is the scaling coefficient predicted based on the charges, dipoles and moments of intertia.

Interaction	r_{com}	k_{ij}^{sapt}	k_{ij}^{pred}
$[C_2C_1Im]^+$–$[NTf_2]^-$	4.314	0.65	0.55

For a more comprehensive overview, the interested reader is kindly referred to GitHub at https://github.com/kirchners-manta/il-benchmark, where a collection of relevant data and input files is provided.

2 Densities of simulation boxes

In table S4 and fig. S1, detailed information on the densities of the MD simulation boxes is presented. The density ρ was well-converged during the averaging process of the equilibration run, as evidenced by the small and almost negligible uncertainties $\Delta\rho$. When comparing this density to the experimental reference data, all systems, except for $\text{MD}^{512\text{-}08}$, exhibit a deviation $\Delta\rho_{\text{exp}}$ of less than 1.5 % from the experimental reference. However, the charge-scaled $\text{MD}^{512\text{-}0.8}$ system demonstrates a larger deviation of −4.41 %. This increased deviation is expected because, while decreasing the charges to enhance the dynamics of the ions, all other parameters of the underlying CL&P[S1–S6] force field remain unchanged.

Table S4: Densities ρ, their uncertainties $\Delta\rho$ from the simulations and experiment (all in $\mathrm{g\,cm^{-3}}$) as well as deviation from the experiment $\Delta\rho_{\mathrm{exp}}$ (in %). The data are visualized in fig. S1.

System	ρ	$\Delta\rho$	$\Delta\rho_{\mathrm{exp}}$
MD^{256}	1.4409	0.0001	−1.43
MD^{512}	1.4411	0.0001	−1.42
MD^{1024}	1.4417	0.0001	−1.38
$\mathrm{MD}^{512\text{-}08}$	1.3974	0.0001	−4.14
NGOLP^{512}	1.4477	0.0001	−0.97
polMD^{256}	1.4553	0.0001	−0.45
polMD^{512}	1.4552	0.0001	−0.46
exp.[S10]	1.4619	0.0001	–

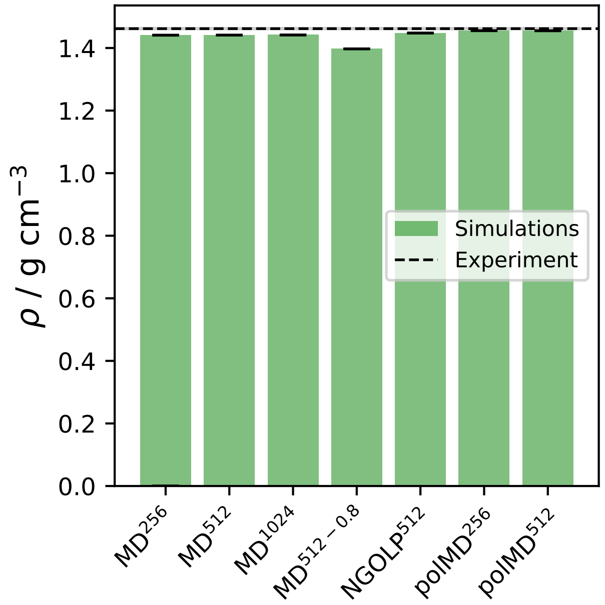

Figure S1: Densities (blue bars) and their uncertainties (black) in comparison to experimental reference data (dashed blue line and gray area for errors) from Ref. S10.

3 Conductivity equations

Here, we provide the precise equations of the various contributions to the Einstein–Helfand ionic conductivity σ_{EH}:

$$\sigma_{+-} = \frac{e^2}{6Vk_\mathrm{B}T} \lim_{\tau\to\infty} \frac{\mathrm{d}}{\mathrm{d}\tau} \left\langle 2 \sum_i^{N_+} \sum_j^{N_-} q_i q_j \cdot \Delta\mathbf{r}_i(t,\tau)\Delta\mathbf{r}_j(t,\tau) \right\rangle_t \quad , \tag{1}$$

$$\sigma_\mathrm{z}^\mathrm{self} = \frac{e^2}{6Vk_\mathrm{B}T} \lim_{\tau\to\infty} \frac{\partial}{\partial\tau} \left\langle \sum_i^{N_\mathrm{z}} q_i^2 \cdot (\Delta\mathbf{r}_i(t,\tau))^2 \right\rangle_t \quad , \tag{2}$$

$$\sigma_\mathrm{zz}^\mathrm{cross} = \frac{e^2}{6Vk_\mathrm{B}T} \lim_{\tau\to\infty} \frac{\partial}{\partial\tau} \left\langle \sum_{i\neq j}^{N_\mathrm{z}} q_i q_j \cdot \Delta\mathbf{r}_i(t,\tau)\Delta\mathbf{r}_j(t,\tau) \right\rangle_t \quad . \tag{3}$$

The above equations use the elementary charge e, the simulation box volume V, the Boltzmann constant k_B, temperature T, and correlation time τ. In addition, N denotes the number of particles (ions), q_i, q_j are the charges of the particles i and j, respectively, z denotes the sign of the ion's charge ($\mathrm{z} \in \{+, -\}$), and $\mathbf{r}_i$ is the time-dependent position of the center of mass (or any other chosen reference point) of ion i. The self terms (eq. (2)) feature a single summation and thus, are denoted by z, while the cross terms, see eq. (3), are denoted by zz to acknowledge the two-fold summation. It is important to note that σ_{+-} is a cross term by definition and hence, there is no corresponding self term.

4 Estimating the uncertainty of the conductivity

As outlined in the main text, the conductivity data calculated from the equations in section 3 is very statistically sensitive, which is visualized in fig. S2 (top right to bottom left panels), where the collective MSDs of the individual trajectories are presented. Various data sets show a strictly nonlinear behavior and thus, hamper a straightforward linear regression to obtain the conductivity. To circumvent this issue, we perform the linear regression on the averaged data, shown in the bottom right panel, as outlined in the Tutorial Part of the main text. Ionic conductivities obtained by that approach are given in detail in table S5, alongside the uncertainties of the error weighted fit.

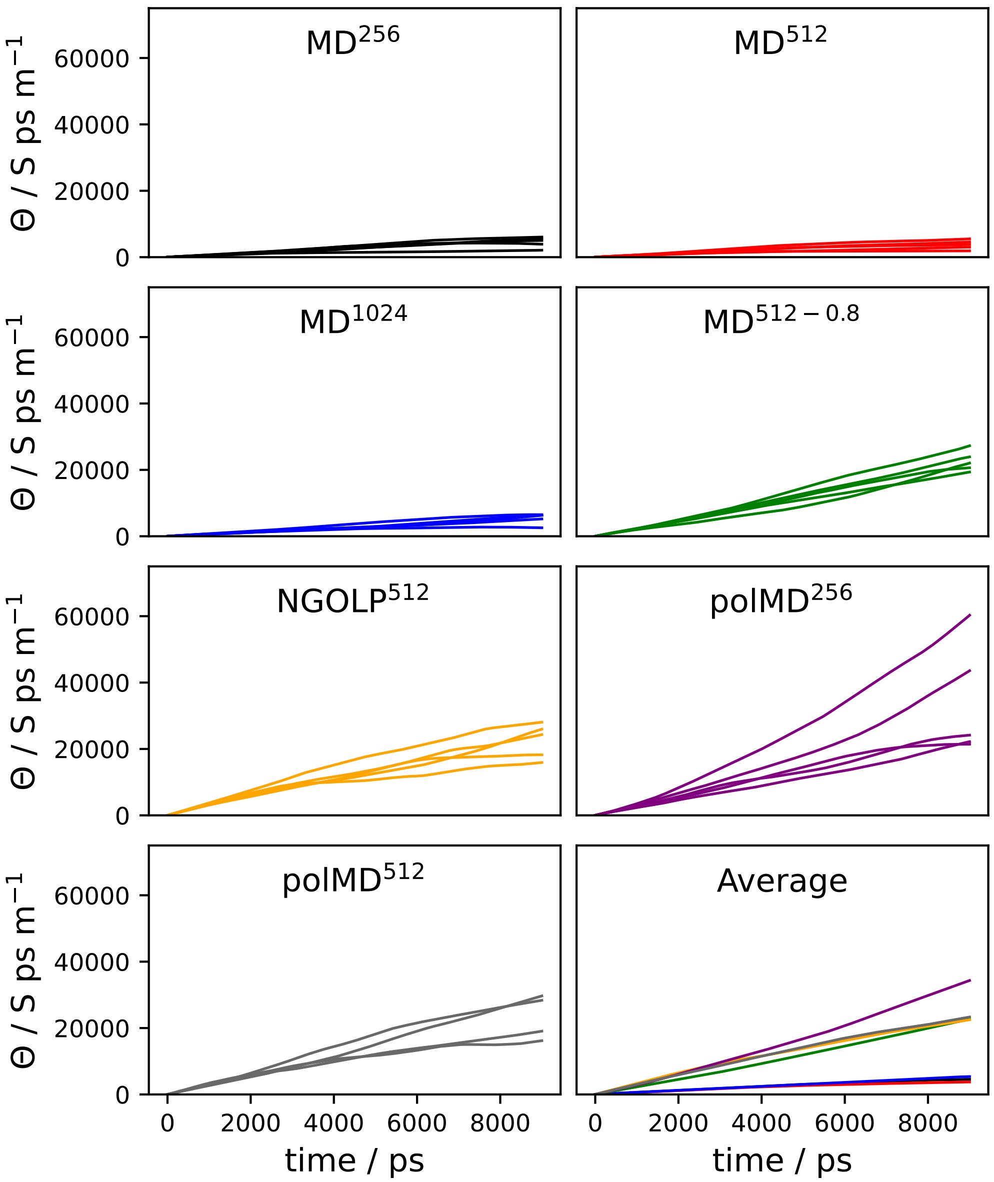

Figure S2: Visualization of the conductivity data sets obtained from all trajectories of the seven systems studied (top right to bottom left panels). The average conductivity data is shown in the bottom right panel.

As outlined in the main text, applying our post-processing routine to the collective MSD of the individual trajectories using a tolerance of 0.1 will not yield any results for some of the trajectories, due to nonlinear behavior shown in fig. S2. For the purpose of demonstration, by increasing the tolerance to 0.2, we were able to obtain ionic conductivities for every trajectory individually. Then, we calculated the mean and standard deviation of the ionic

conductivities for every system and list them as $\sigma_{\mathrm{NE}}^{\mathrm{ave}}$, $\Delta\sigma_{\mathrm{NE}}^{\mathrm{ave}}$, $\sigma_{\mathrm{EH}}^{\mathrm{ave}}$ and $\Delta\sigma_{\mathrm{EH}}^{\mathrm{ave}}$ in table S5. It should be noted, that these uncertainties represent the uncertainties in the set of trajectories and consequently, are not comparable to those obtained from the error weighted fit ($\Delta\sigma_{\mathrm{NE}}$, $\Delta\sigma_{\mathrm{EH}}$).

The spread of the collective MSD in the individual trajectories is reflected in the large values of $\Delta\sigma_{\mathrm{EH}}^{\mathrm{ave}}$, which make up for 45 % in case of MD^{512} and around 15 % to 25 % for the other systems. From that observation, we conclude that in future studies, instead of five independent trajectories per system, it is recommended to perform 15-20 replica simulations.

Table S5: Ionic conductivities calculated using the Nernst–Einstein (σ_{NE}) and Einstein–Helfand (σ_{EH}) formalisms and their uncertainties obtained from the averaged collective MSD in comparison to the ionic conductivities ($\sigma_{\mathrm{NE}}^{\mathrm{ave}}$, $\sigma_{\mathrm{EH}}^{\mathrm{ave}}$)and uncertainties obtained from the individual trajectories and subsequent averaging (all in $\mathrm{S\,m^{-1}}$). Experimentally derived reference data are also given (refer to section 5).

System	σ_{NE}	$\Delta\sigma_{\mathrm{NE}}$	$\sigma_{\mathrm{NE}}^{\mathrm{ave}}$	$\Delta\sigma_{\mathrm{NE}}^{\mathrm{ave}}$	σ_{EH}	$\Delta\sigma_{\mathrm{EH}}$	$\sigma_{\mathrm{EH}}^{\mathrm{ave}}$	$\Delta\sigma_{\mathrm{EH}}^{\mathrm{ave}}$
MD^{256}	0.6669	0.0002	0.6599	0.0242	0.5832	0.0002	0.6538	0.1476
MD^{512}	0.6680	0.0002	0.6554	0.0231	0.5618	0.0002	0.4850	0.2187
MD^{1024}	0.6766	0.0001	0.6706	0.0047	0.6051	0.0001	0.6025	0.1531
$\mathrm{MD}^{512\text{-}08}$	2.8908	0.0001	2.8929	0.0216	2.6626	0.0004	2.4688	0.5985
NGOLP^{512}	4.1785	0.0001	4.1238	0.0876	3.2875	0.0001	2.8690	0.4211
polMD^{256}	3.8751	0.0001	3.8711	0.0897	3.5217	0.0005	3.1514	0.5771
polMD^{512}	4.3049	0.0005	4.2357	0.0856	2.2940	0.0009	2.8637	0.8082
Ref.	–	–	–	–	3.0057	0.1119	–	–

Additionally, we estimate the **maximum possible** error introduced by the averaging. As we average the collective MSD, the value of every data point of the averaged set (depicted in the bottom right panel of fig. S2) carries a standard deviation. This standard deviation (usually referenced as σ but here labeled Ω to avoid confusion) is used in the following procedure.

As the collective MSD Θ in increases (approximately) linear with the correlation depth τ, the standard deviation is assumed to increase linearly as well. In consequence, the relation $\Omega(\tau_1) < \Omega(\tau_2)$ is fulfilled for $\tau_2 \gg \tau_1$. From our fitting routine (described in the main text), we usually obtain linear regimes $\tau_1 < \tau < \tau_2$ that obey the latter condition. Therefore,

we can define upper and lower bounds σ_{up}, σ_{low} in between which the conductivity will be located. The slope of the line connecting the two points $\Theta(\tau_1) - \Omega(\tau_1)$ and $\Theta(\tau_2) + \Omega(\tau_2)$ serves as an upper bound for the conductivity. Accordingly, the slope of the line connecting $\Theta(\tau_1) + \Omega(\tau_1)$ and $\Theta(\tau_2) - \Omega(\tau_2)$ serves as the lower bound. This is visualized in fig. S3.

The black solid line is the averaged Θ data set of an arbitrary system, while the standard deviation is indicated by the gray shaded area. Black dashed lines are located at τ_1 and τ_2 and indicate the fitting interval. It is evident that the slopes of the red lines are clearly different from the slope of the black solid line, and can serve as upper and lower bounds for σ. Precisely, we calculate the bounds as follows:

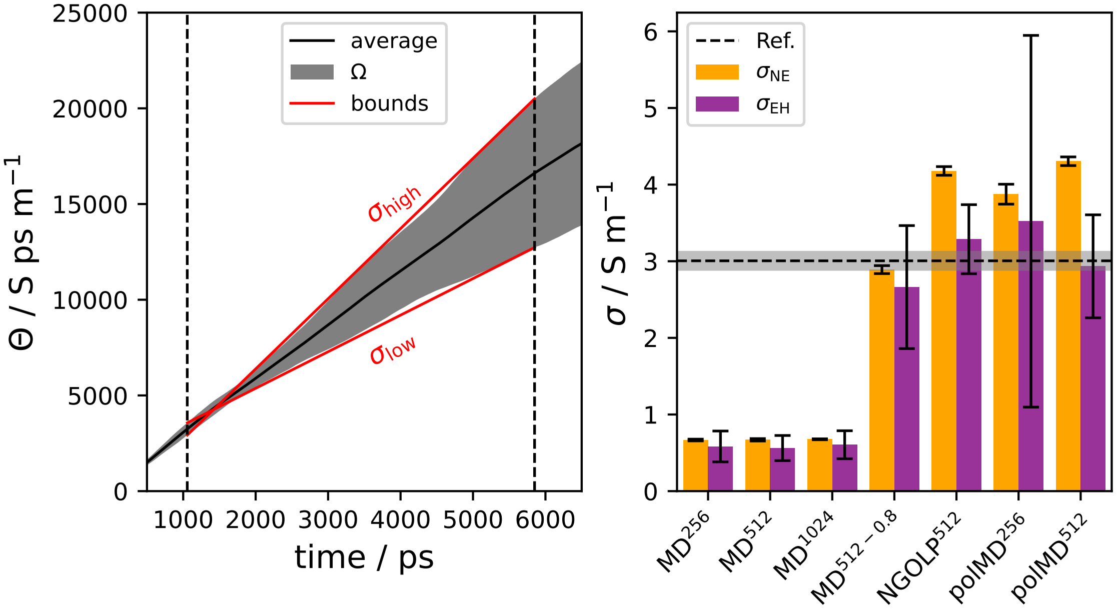

Figure S3: **Left**: Illustration of the estimation of the maximum uncertainty of the conductivity at the example of an arbitrary data set. The panel shows the averaged collective MSD Θ (black line) and the interval of one standard deviation Ω (gray shaded area). The black dashed lines at τ_1, τ_2 indicate the linear regime identified by our routine (see main text) and the slope of the red lines correspond to the lower and upper bounds of the conductivity σ_{low}, σ_{high}. **Right**: Visualization of the maximum uncertainty of σ_{NE} (orange bars) and σ_{EH} (purple bars) found in our simulations, in comparison to reference data (gray shaded area).

$$\sigma_{\mathrm{low}} = \frac{[\Theta(\tau_2) - \Omega(\tau_2)] - [\Theta(\tau_1) + \Omega(\tau_1)]}{\tau_2 - \tau_1}, \tag{4}$$

$$\sigma_{\text{hi}} = \frac{[\Theta(\tau_2) + \Omega(\tau_2)] - [\Theta(\tau_1) - \Omega(\tau_1)]}{\tau_2 - \tau_1}. \tag{5}$$

Then, we obtain our estimation of the maximum uncertainty of the conductivity as the difference between these two bounds:

$$\Delta\sigma = \frac{\sigma_{\text{hi}} - \sigma_{\text{low}}}{2}. \tag{6}$$

At this point, it is important to note that by adhering to this procedure, we can estimate the **maximum** error of the conductivity introduced by our routine. The "true" uncertainty may in fact be smaller.

5 Calculated and reference conductivities

In addition to the visualization provided in the main text, we provide a table with experimentally derived references for the ionic conductivity in table S6. For visualization in the corresponding figure in the main text, we use the mean and the standard deviation of the values listed

Table S6: Reference ionic conductivities, σ_{exp} as well as their uncertainties, $\Delta\sigma_{\text{exp}}$ (in S m^{-1}). Values at 350 K are obtained by a Vogel–Fulcher–Tamman scheme (Refs. S11–S16) or a third-order polynomial (Ref. S17).

Ref.	σ_{exp}	$\Delta\sigma_{\text{exp}}$
Every *et al.*[S11]	3.284	0.323
Makino *et al.*[S12]	2.956	0.114
Schreiner *et al.*[S13]	3.027	0.024
Tokuda *et al.*[S14]	2.904	0.114
Ramenskaya *et al.*[S15]	3.010	0.183
Tokuda *et al.*[S16]	2.924	0.284
Calado *et al.*[S17]	2.987	0.117

6 Local dynamics

In tables S7 and S8, we provide full information on the calculated local dynamics, showing all seven systems studied.

Table S7: Ion pair (IP) and ion cage (CG) lifetimes $\mathcal{T}$ of $[NTf_2]^-$ around $[C_2C_1Im]^+$ and their corresponding uncertainties (all in ps), for all 512 ion pair systems. Correlation functions were calculated in continuous (C) and intermittent (I) fashion.

System	$\mathcal{T}_C^{IP}$	$\Delta\mathcal{T}_C^{IP}$	$\mathcal{T}_I^{IP}$	$\Delta\mathcal{T}_I^{IP}$	$\mathcal{T}_C^{CG}$	$\Delta\mathcal{T}_C^{CG}$	$\mathcal{T}_I^{CG}$	$\Delta\mathcal{T}_I^{CG}$
MD^{256}	20.4	0.4	1354	11	1257.0	2.5	7253	11
MD^{512}	20.4	0.5	1201	11	1253.2	3.5	7842	17
MD^{1024}	20.1	0.4	1237	13	1238.7	3.1	7842	15
$MD^{512\text{-}08}$	10.8	0.1	439	4	375.5	0.8	2150	7
$NGOLP^{512}$	8.5	0.5	286	3	301.3	0.7	1211	5
$polMD^{256}$	9.6	0.5	327	4	312.5	0.6	1712	5
$polMD^{512}$	9.6	0.1	296	2	289.9	0.4	1641	4

Table S8: Reorientation times τ of the cations (in ps). Three different vectors, oriented perpendicular to each other (cf. Fig. 1 of the main text), were considered in reorientation analyzes: $\vec{a}_{C2,H}$ connects C2 and the adjacent hydrogen atom, $\vec{a}_{N1,N2}$ connects the two nitrogen atoms, and $\vec{a}_{C2,C3,C4}$ stands perpendicular to the plane created by the respective atoms (i.e., the ring plane) The reader is referred to fig. 1 of the manuscript to see the exact labelling of atoms of the cation.

	$\vec{a}_{C2,H}$		$\vec{b}_{N1,N2}$		$\vec{c}_{C2,C3,C4}$	
System	$\mathcal{T}$	$\Delta\mathcal{T}$	$\mathcal{T}$	$\Delta\mathcal{T}$	$\mathcal{T}$	$\Delta\mathcal{T}$
MD^{256}	83.9	0.3	337.1	0.7	95.2	0.3
MD^{512}	72.2	0.5	318.2	1.1	84.8	0.4
MD^{1024}	67.7	0.3	367.8	0.6	86.9	0.4
$MD^{512\text{-}08}$	24.9	0.1	97.7	0.1	30.4	0.1
$NGOLP^{512}$	28.0	0.1	102.4	0.1	27.6	0.1
$polMD^{256}$	25.8	0.1	86.2	0.1	29.5	0.1
$polMD^{512}$	24.6	0.1	73.8	0.1	28.9	0.1

References

(S1) Pádua, A. A. H.; Canongia Lopes, J. N.; Deschamps, J. Modeling Ionic Liquids Using a Systematic All-Atom Force Field. *J. Phys. Chem. B* **2004**, *108*, 2038–2047.

(S2) Pádua, A. A. H.; Canongia Lopes, J. N.; Deschamps, J. Modeling Ionic Liquids Using a Systematic All-Atom Force Field. *J. Phys. Chem. B* **2004**, *108*, 11250–11250.

(S3) Pádua, A. A. H.; Canongia Lopes, J. N. Molecular Force Field for Ionic Liquids Composed of Triflate or Bistriflylimide Anions. *J. Phys. Chem. B* **2004**, *108*, 16893–16898.

(S4) Pádua, A. A. H.; Canongia Lopes, J. N. Molecular Force Field for Ionic Liquids III: Imidazolium, Pyridinium, and Phosphonium Cations; Chloride, Bromide, and Dicyanamide Anions. *J. Phys. Chem. B* **2006**, *110*, 19586–19592.

(S5) Pádua, A. A. H.; Canongia Lopes, J. N.; Shimizu, K. Molecular Force Field for Ionic Liquids IV: Trialkylimidazolium and Alkoxycarbonyl-Imidazolium Cations; Alkylsulfonate and Alkylsulfate Anions. *J. Phys. Chem. B* **2008**, *112*, 5039–5046.

(S6) Pádua, A. A. H.; Shimizu, K.; Almantariotis, D.; Gomes, M. F. C.; Canongia Lopes, J. N. Molecular Force Field for Ionic Liquids V: Hydroxyethylimidazolium, Dimethoxy-2- Methylimidazolium, and Fluoroalkylimidazolium Cations and Bis(Fluorosulfonyl)Amide, Perfluoroalkanesulfonylamide, and Fluoroalkylfluorophosphate Anions. *J. Phys. Chem. B* **2010**, *114*, 3592–3600.

(S7) Neumann, J.; Golub, B.; Odebrecht, L.-M.; Ludwig, R.; Paschek, D. Revisiting Imidazolium Based Ionic Liquids: Effect of the Conformation Bias of the [NTf2] Anion Studied by Molecular Dynamics Simulations. *J. Chem. Phys.* **2018**, *148*, 193828.

(S8) Goloviznina, K.; Canongia Lopes, J. N.; Costa Gomes, M.; Pádua, A. A. H. Transferable, Polarizable Force Field for Ionic Liquids. *J. Chem. Theory Comput.* **2019**, *15*, 5858–5871.

(S9) Goloviznina, K.; Gong, Z.; Costa Gomes, M. F.; Pádua, A. A. H. Extension of the CL&Pol Polarizable Force Field to Electrolytes, Protic Ionic Liquids, and Deep Eutectic Solvents. *J. Chem. Theory Comput.* **2021**, *17*, 1606–1617.

(S10) Gardas, R. L.; Freire, M. G.; Carvalho, P. J.; Marrucho, I. M.; Fonseca, I. M. A.; Ferreira, A. G. M.; Coutinho, J. A. P. PpT Measurements of Imidazolium-Based Ionic Liquids. *J. Chem. Eng. Data* **2007**, *52*, 1881–1888.

(S11) Every, H. A.; G. Bishop, A.; R. MacFarlane, D.; Orädd, G.; Forsyth, M. Transport Properties in a Family of Dialkylimidazolium Ionic Liquids. *Phys. Chem. Chem. Phys.* **2004**, *6*, 1758–1765.

(S12) Makino, T.; Kanakubo, M.; Masuda, Y.; Umecky, T.; Suzuki, A. CO2 Absorption Properties, Densities, Viscosities, and Electrical Conductivities of Ethylimidazolium and 1-Ethyl-3-Methylimidazolium Ionic Liquids. *Fluid Ph. Equilibria* **2014**, *362*, 300–306.

(S13) Schreiner, C.; Zugmann, S.; Hartl, R.; Gores, H. J. Fractional Walden Rule for Ionic Liquids: Examples from Recent Measurements and a Critique of the So-Called Ideal KCl Line for the Walden Plot. *J. Chem. Eng. Data* **2010**, *55*, 1784–1788.

(S14) Tokuda, H.; Hayamizu, K.; Ishii, K.; Susan, M. A. B. H.; Watanabe, M. Physicochemical Properties and Structures of Room Temperature Ionic Liquids. 2. Variation of Alkyl Chain Length in Imidazolium Cation. *J. Phys. Chem. B* **2005**, *109*, 6103–6110.

(S15) Ramenskaya, L. M.; Grishina, E. P.; Kudryakova, N. O. Physicochemical Features of Short-Chain 1-Alkyl-3-Methylimidazolium Bis(Trifluoromethylsulfonyl)-Imide Ionic Liquids Containing Equilibrium Water Absorbed from Air. *J. Mol. Liq.* **2018**, *272*, 759–765.

(S16) Tokuda, H.; Tsuzuki, S.; Susan, M. A. B. H.; Hayamizu, K.; Watanabe, M. How Ionic Are Room-Temperature Ionic Liquids? An Indicator of the Physicochemical Properties. *J. Phys. Chem. B* **2006**, *110*, 19593–19600.

(S17) Calado, M. S.; Diogo, J. C. F.; Correia da Mata, J. L.; Caetano, F. J. P.; Visak, Z. P.; Fareleira, J. M. N. A. Electrolytic Conductivity of Four Imidazolium-Based Ionic Liquids. *Int. J. Thermophys.* **2013**, *34*, 1265–1279.
